# Comparative Transcriptomic and Lipidomic Analysis of Amphotericin B‑Susceptible and Resistant Clinical Isolates of *Malassezia* spp

**DOI:** 10.1021/acsomega.6c09344

**Published:** 2026-10-27

**Authors:** Juan Camilo Galvis-Marín, Fredy Alexander Tabares-Villa, Augusto Zuluaga-Vélez, Paola Rojas-Estevez, Mónica P. Cala, Adriana Marcela Celis-Ramírez, Juan Carlos Sepúlveda-Arias

**Affiliations:** † Grupo Infección e Inmunidad, Facultad Ciencias de la Salud, Universidad Tecnológica de Pereira, Carrera 27 #10-02 Barrio Álamos, Pereira, Risaralda 660003, Colombia; ‡ Grupo de Investigación Genómica de Microorganismos Emergentes, Dirección de Investigación, Instituto Nacional de Salud, Avenida Calle 26 #51-20, Zona 6 CAN, Bogotá, DC 111321, Colombia; § Metabolomics Core Facility (MetCore), Vicepresidency for Research, 27991 Universidad de los Andes, Cra 1 #18A-12, Bogotá, DC 111711, Colombia; ∥ Grupo de Investigación Celular y Molecular de Microorganismos Patógenos (CeMoP), Facultad de Ciencias, 27991 Universidad de los Andes, Cra 1 #18A-12, Bogotá, DC 111711, Colombia

## Abstract

*Malassezia* spp. are lipid-dependent skin commensal yeasts associated with superficial infections and fungemia. Amphotericin B remains a key treatment for systemic infections despite its toxicity and decreased susceptibility. This study characterized amphotericin B susceptibility profiles and identified transcriptomic and lipidomic signatures of polyene resistance in clinical *Malassezia* isolates. Clinical isolates of *M*. *sympodialis* and *M*. *furfur*, and reference strains (CBS 7222 and CBS 1878), were evaluated using the modified M27-A3 CLSI protocol for MICs and subinhibitory concentrations. One isolate per species was susceptible (MIC 0.5–2.0 μg/mL), while another was resistant (MIC ≥ 16 μg/mL). Transcriptomes generated by *Oxford Nanopore* sequencing were analyzed using DESeq2 (|log2FC|>2, *p* < 0.05). Comparisons between susceptible and resistant isolates revealed few Differentially Expressed Genes (DEGs) that were not enriched for ergosterol biosynthesis. Interaction hubs indicated roles in nucleotide homeostasis, translation, chromatin regulation, and stress pathways. Lipidomic analysis by HPLC-QTOF-MS detected approximately 200 lipid species, including 55 Differential Lipid Molecules (DLMs) for *M*. *sympodialis* and 63 for *M*. *furfur*. Although global class abundances remained unchanged, resistant clinical isolates showed differences in the relative abundance of selected lipid species, with increased levels of long-chain fatty acids, diacylglycerols, and ceramides. Resistant *M*. *sympodialis* showed higher cholesterol levels. Integrated analysis separated susceptible and resistant groups, with DEGs–DLMs correlations highlighting networks in membrane organization, stress tolerance, and metabolism. These findings suggest that amphotericin B resistance may be associated with metabolic alterations and changes in membrane lipid composition, supporting the use of multiomics markers for detection and therapeutic strategies.

## Graphical abstract

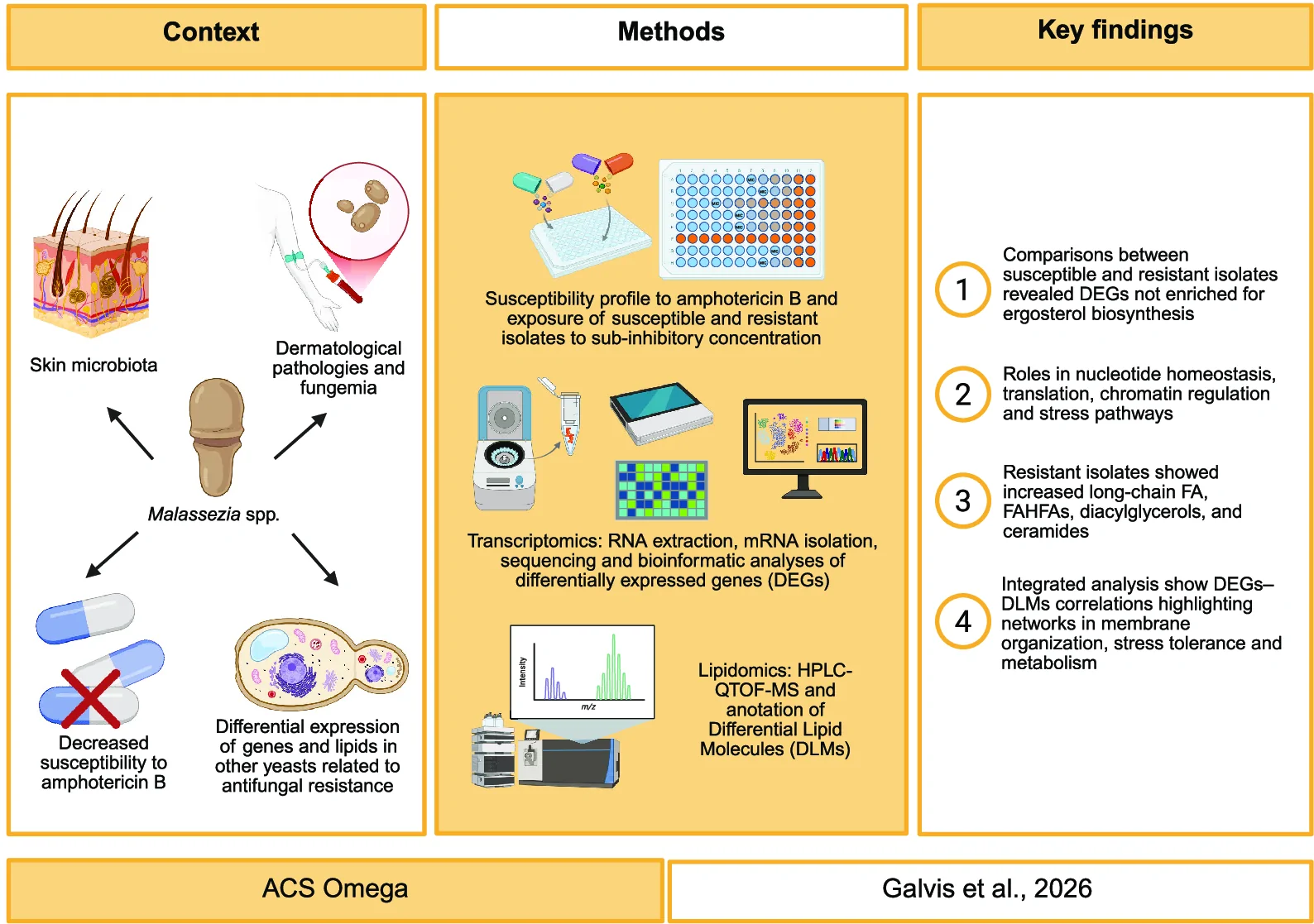

## Introduction

*Malassezia* comprises lipid-dependent yeasts that are skin commensals in both humans and animals and can become pathogenic under certain predisposing conditions. Currently, 23 species are recognized within the genus, of which *M*. *globosa*, *M*. *restricta*, *M*. *sympodialis*, and *M*. *furfur* are the main species isolated from normal human skin mycobiota.^1–3^ These yeasts are the etiological agents of the superficial mycosis pityriasis versicolor and are associated with other dermatological conditions, such as seborrheic dermatitis, psoriasis, and atopic dermatitis.^4^ They can also be opportunistic pathogens and cause fungemia in immunocompromised individuals.^5^ Recently, these yeasts have been linked to the pathogenesis of several chronic human diseases, including cystic fibrosis, rhinosinusitis, asthma, Crohn’s disease, malignant neoplasms of the colon, rectum, and pancreas, and neurodegenerative diseases.^6^ Most invasive infections are caused by *M*. *furfur* and *M*. *pachydermatis*,^4^ however, some cases of fungemia due to *M*. *sympodialis* have also been reported in the medical literature.^7^ Polyenes, such as amphotericin B, are used to treat these fungal infections. Amphotericin B is a fungicidal drug that remains the drug of choice for treating invasive fungal infections, including those caused by *Malassezia* spp., despite its toxic side effects.^8^ In addition, reports of decreased susceptibility to this antifungal agent exist, and the molecular mechanisms underlying polyene resistance remain poorly understood.^9^

Polyene resistance mechanisms include alterations in sterols, phospholipids, and fatty acids (FA) in the cell membrane. Most of this knowledge comes from studies on *Saccharomyces cerevisiae*, *Candida* spp., and *Aspergillus* spp.^10,11^ Gene expression analyses have shown the upregulation of the efflux pump genes *PDR5* and *PDR10* ^12^ in azole-resistant *M*. *restricta* and *M*. *furfur* isolates. However, gene expression in *Malassezia* spp. isolates, susceptible and resistant to amphotericin B, remains undetermined in transcriptomic analyses.

Considering the relevance of lipid metabolism and its applications, lipidomics has emerged within omics, and its application, sensitivity, and reliability have increased with advances in mass spectrometry techniques.^13,14^ Lipidomic analyses have been performed in fungal pathogens such as *Cryptococcus* spp., *Candida* spp., and *Paracoccidioides brasiliensis* to investigate their virulence, antifungal resistance, and novel antifungal targets.^15,16^ A recent study examined the lipid composition of *C*. *auris* and showed a correlation with antifungal resistance. They compared lipidomics profiles of susceptible and multidrug-resistant isolates using mass spectrometry. The resistant isolate showed increased phosphoglycerides (phosphatidylcholine (PC), phosphatidylglycerol (PG), phosphatidylserine (PS), phosphatidylinositol (PI), and phosphatidylethanolamine (PE)), odd-chain-containing lipids, 16:1-diacylglycerols (DAG) and 16:0-DAG, and depleted 18:1-triacylglycerols (TAG) and 18:0-TAG. Unsaturated phosphoglyceride levels were higher in the resistant isolate, resulting in a higher unsaturation index, highlighting modulation of membrane lipid homeostasis in resistant *C*. *auris*.^17^ Since *Malassezia* spp. genomes lack FA synthesis genes, Triana et al. reconstructed lipid synthesis pathways of *M*. *globosa*, *M*. *sympodialis*, *M*. *pachydermatis*, and *M*. *furfur*, finding flux differences in steroid production in *M*. *furfur* and butanoate metabolism in *M*. *pachydermatis*, as well as palmitic acid assimilation defects in *M*. *globosa*, *M*. *sympodialis*, and *M*. *pachydermatis*. Physiological characterization validated these predictions, showing the value of metabolic network reconstructions for understanding *Malassezia* metabolism.^18^

Lipidomic analysis of a subset of *Malassezia* species was recently performed, along with gene prediction to detect lipid-metabolism proteins.^19^ The study identified 18 lipid classes and 428 lipid compounds, the most common of which were TAG, sterols, DAG, FA, PC, PE, ceramides, cholesteryl esters, sphingomyelin, acylcarnitine, and lysophospholipids. *M*. *furfur* and *M*. *pachydermatis* had low levels of cholesteryl esters, while *M*. *globosa*, *M*. *restricta*, and *M*. *sympodialis* had undetectable traces of these components. However, membrane lipid composition in *Malassezia* spp. isolates that are susceptible and resistant to amphotericin B have not yet been compared.

Combining transcriptomic and lipidomic analyses offers a powerful approach to comprehensively characterizing resistance phenotypes. Transcriptomic profiling facilitates the detection of differential gene expression in pathways related to sterol biosynthesis, lipid transport, cell wall organization, stress responses, and signaling, all of which may contribute to antifungal resistance mechanisms. Complementarily, lipidomic analyses provide insight into changes in membrane lipid composition, abundance, and spatial organization. Such alterations may affect the interaction between amphotericin B and membrane sterols or modify membrane structure, ultimately diminishing the drug’s antifungal activity. Therefore, this study aimed to evaluate the susceptibility profiles of clinical isolates of *Malassezia* spp. to amphotericin B and to characterize their transcriptomes and lipidomes to identify gene expression patterns and lipidomic signatures associated with polyene resistance. This integrative approach will provide a solid foundation for developing targeted therapeutic strategies and expanding our understanding of *Malassezia* biology and its adaptation to antifungal pressure.

## Results and Discussion

An essential factor for the effective treatment of mycoses may be the early detection of infections caused by pathogens with a high potential to develop antifungal resistance. Early identification of these strains would enable precise treatment, including appropriate selection and dosing of antifungal agents.^20^ In the present study, transcriptomic and lipidomic analyses were performed on amphotericin B susceptible and resistant *Malassezia* spp. isolates to elucidate the resistance mechanisms of these yeasts to polyene antifungals.

### Susceptibility Profile of *Malassezia* spp. Isolates

The Minimum Inhibitory Concentration (MIC) of amphotericin B and subinhibitory concentrations were determined using broth microdilution and spot assays, respectively. We determined the amphotericin B susceptibility profile for 26 clinical isolates and two reference strains, with MICs assessed in three independent replicates. Because standardized clinical breakpoints for amphotericin B are not available for *Malassezia* spp., isolates were classified according to the MIC_50_/MIC_90_-based criterion proposed by Cafarchia et al.^21^ Based on this criterion, we operationally categorized isolates as susceptible or resistant. From this larger susceptibility collection, one susceptible and one resistant clinical isolate of each species were selected for the transcriptomic and lipidomic analyses, together with the corresponding resistant reference strain (Table 1).

**Table 1:** *Malassezia* spp. Isolates and Reference Strains According to Origin, Underlying Pathology, Molecular Identification and Susceptibility Profile to Amphotericin B^a^

**code**	**origin**	**pathology**	**molecular identification**	**MIC**(μg/mL)	**interpretation** ^a^	**subinhibitory concentration**(μg/mL)
20PV	human skin	pityriasis versicolor	*M*. *sympodialis*	0.5–1.0	S	0.5
1DA	human skin	atopic dermatitis	*M*. *sympodialis*	≥16.0	R	16.0
3DA	human skin	atopic dermatitis	*M*. *furfur*	1.0–2.0	S	1.0
14DS HIV	human skin	seborrheic dermatitis and HIV	*M*. *furfur*	≥16.0	R	16.0
CBS 7222	human external ear	none	*M*. *sympodialis*	≥16.0	R	16.0
CBS 1878	human scalp	dandruff	*M*. *furfur*	≥16.0	R	16.0

a Susceptible (S) = MIC isolate ≤ MIC_50_; Dose-Dependent susceptible (DDS) = MIC_50_ < MIC isolate ≤ MIC_90_; Resistant (R) = MIC isolate > MIC_90_, according to the criterion proposed by Cafarchia et al.^21^ MIC_50_ and MIC_90_ were calculated from a larger susceptibility data set comprising 26 clinical isolates and two reference strains. These categories are operational phenotypic classifications and do not represent established clinical breakpoints for amphotericin B in *Malassezia* spp.

Most invasive infections are caused by *M*. *furfur* and *M*. *pachydermatis*. However, the medical literature also reports cases of fungemia due to *M*. *sympodialis*.^4,7^ In the present study, *M*. *sympodialis* was included because clinically characterized isolates with contrasting amphotericin B susceptibility phenotypes were available in our collection, whereas resistant *M*. *pachydermatis* isolates were not available for comparative analysis. Thus, the species selection was primarily determined by the availability of isolates with the required susceptibility phenotypes and was not intended to represent the species distribution of invasive *Malassezia* infections.

### Transcriptomic Analysis

To perform transcriptomic analysis of amphotericin B resistance genes, we extracted total RNA from the isolates and reference strains, with and without exposure to subinhibitory concentrations of amphotericin B. Bioinformatics analyses generated 176686 and 216211 raw reads for *M*. *sympodialis* and *M*. *furfur*, respectively. Before filtering, *M*. *sympodialis* reads had a mean length of 588.6 bp, a median length of 475 bp, and an N50 of 661 bp, whereas *M*. *furfur* reads had a mean length of 588.4 bp, a median length of 478 bp, and an N50 of 666 bp. Mean Phred quality scores were 14.4 and 14.3 for *M*. *sympodialis* and *M*. *furfur*, respectively. Reads with quality scores above Q20 accounted for 16.5% (15.9 Mb) of the *M*. *sympodialis* data set and 16.2% (19.2 Mb) of the *M*. *furfur* data set, while more than 99.7% of reads in both data sets exceeded Q10 quality (Supporting Table S1). Adapter trimming and quality filtering resulted in minimal data loss, while overall read length distributions and quality metrics remained virtually unchanged after preprocessing (Supporting Table S2). The sequencing reads generated in this study were deposited in the NCBI database Sequence Read Archive under BioProject accession number PRJNA1353145 (https://dataview.ncbi.nlm.nih.gov/object/PRJNA1353145?reviewer=ssu61194dgmd93grngb9vek8hu).

Filtered reads were aligned against the corresponding reference genomes using Minimap2. The reference genome ASM34930v2 was used to quantify gene expression in *M*. *sympodialis*. A size of 7.7 Mb characterizes this genome and contains 3517 genes, of which 3318 are protein-coding. When performing the gene count matrix, 3318 genes showed nonzero expression, indicating that at least one read aligned to those genes was detected in some samples. The reference genome ASM993813v1 was used to quantify gene expression in *M*. *furfur*. A size of 8.3 Mb characterizes this genome, which contains 4601 genes, of which 4533 are protein-coding genes. The gene count matrix yielded 4394 genes with nonzero expression. Mapping rates were consistently high across samples, ranging from 75.3 to 96.7% in *M*. *sympodialis* and from 86.3 to 98.7% in *M*. *furfur* (Supporting Table S2), supporting the reliability of downstream transcriptomic analyses.

We performed correlation analyses based on raw gene count data to compare resistant *Malassezia* spp. isolates exposed and unexposed to amphotericin B (Figure S1A and S1B). Interestingly, exposure to subinhibitory amphotericin B did not result in a major global transcriptional shift in the resistant isolates, as the exposed and unexposed samples retained highly similar overall transcriptomic profiles. The data suggest that, under the experimental conditions used here, amphotericin B exposure was associated with relatively restricted transcriptional changes superimposed on a broadly conserved transcriptomic state. These results also suggest that the resistant phenotype may reflect pre-existing membrane configuration rather than acute stress adaptation. Such constitutive resistance is consistent with the mechanism of action of amphotericin B, which requires direct interaction with ergosterol to form pores. However, the possibility that tolerance is attributable to genetic resistance cannot be excluded.^22^

Given the absence of major transcriptional differences associated with amphotericin B exposure, subsequent analyses focused on constitutive transcriptional differences between phenotypically susceptible and resistant isolates. For differential expression analyses of each species, resistant groups included both the clinical isolate and the reference strain, whereas susceptible groups comprised the clinical isolate.

PCA was performed to explore transcriptional differences between amphotericin B-susceptible and resistant isolates. In *M*. *sympodialis*, PC1 and PC2 explained 29 and 15% of the total variance, respectively (Figure 1A). In *M*. *furfur*, they accounted for 17 and 15% of the variance, respectively (Figure 1C). In *M*. *sympodialis*, the susceptible samples showed considerable dispersion, including one sample that was separated from the remaining susceptible samples along PC2. Although some degree of separation between susceptible and resistant samples was observed along PC1, the groups did not form completely distinct clusters. In *M*. *furfur*, the distribution showed a more evident separation between susceptible and resistant samples, although some within-group variability was also observed. To assess whether the dispersion observed among the *M*. *sympodialis* susceptible samples could be related to an evident sequencing or mapping-quality outlier, alignment statistics were examined (Supporting Table S2). The three susceptible samples exposed to amphotericin B showed mapping rates ranging from 78.89 to 81.78%, while the three corresponding unexposed samples ranged from 82.46 to 84.55%. These values did not reveal an evidently anomalous mapping rate among the susceptible replicates that could readily account for the PCA dispersion, so the available quality metrics do not allow us to establish the biological or technical cause of the separated sample. The observed dispersion may therefore reflect sample-level heterogeneity that cannot be resolved from the present data set.

**Figure 1:**
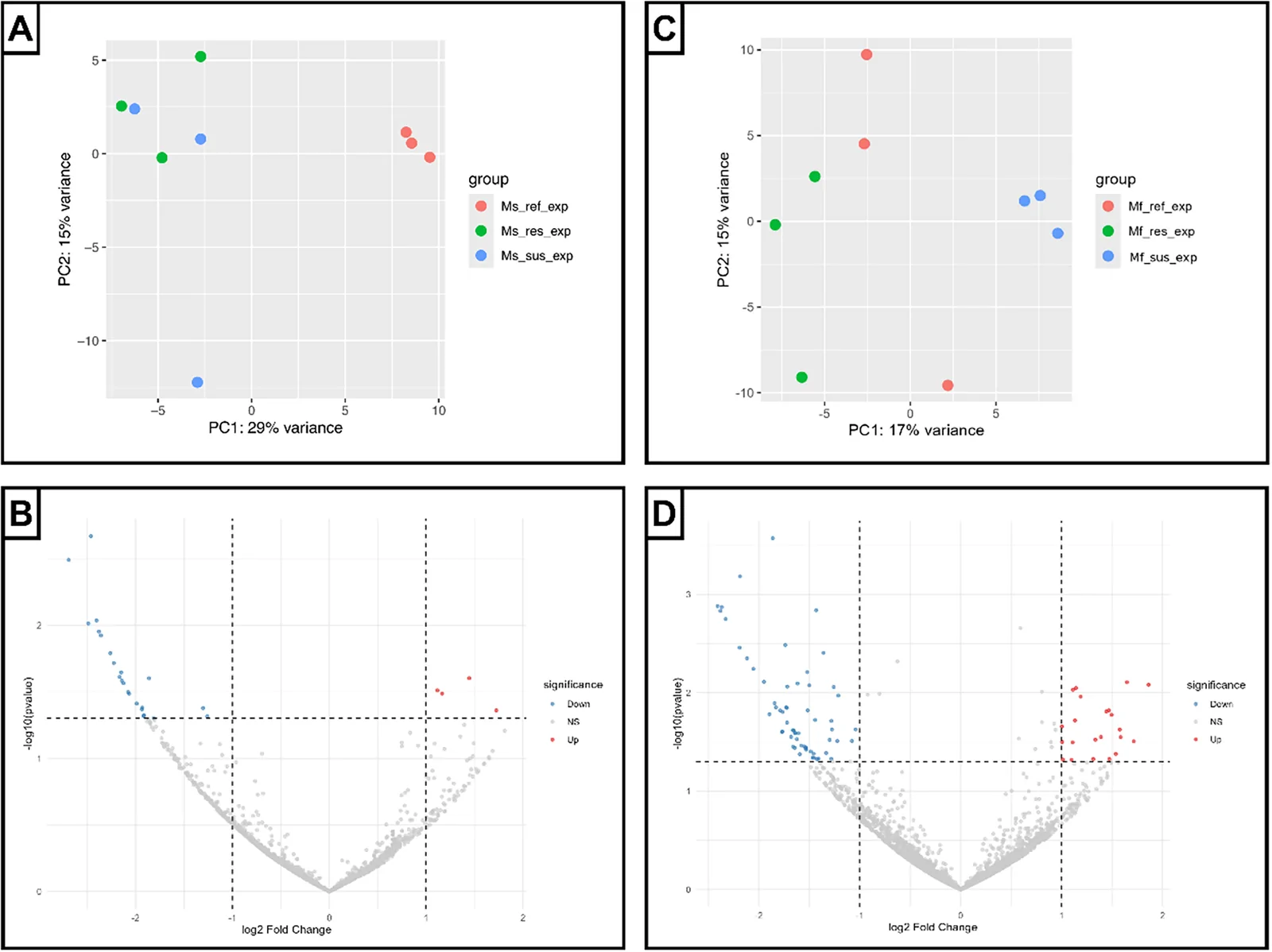
(A) PCA showing global transcriptional differences between amphotericin B-susceptible and resistant *M*. *sympodialis* isolates. Red (Ms_ref_exp): *M*. *sympodialis* CBS 7222, green (Ms_res_exp): *M*. *sympodialis* resistant, blue (Ms_sus_exp): *M*. *sympodialis* susceptible. (B) Volcano plot showing genes upregulated (red) and downregulated (blue) in the comparison of susceptible and resistant *M*. *sympodialis*. (C) PCA showing global transcriptional differences between amphotericin B-susceptible and resistant *M*. *furfur* isolates. Red (Mf_ref_exp): *M*. *furfur* CBS 1878, green (Mf_res_exp): *M*. *furfur* resistant, blue (Mf_sus_exp): *M*. *furfur* susceptible. (D) Volcano plot showing genes upregulated (red) and downregulated (blue) in the comparison of susceptible and resistant *M*. *furfur*.

Therefore, the PCA should not be interpreted as demonstrating consistent transcriptome-wide organization according to amphotericin B susceptibility. These patterns suggest that susceptibility-associated transcriptional differences may contribute to sample-level variation, but they may also reflect isolate-specific biological heterogeneity. In fact, the susceptibility phenotype was not fully independent of isolate background in the present experimental design. In each species, the susceptible phenotype was represented by a single clinical isolate, whereas the resistant phenotype included a clinical isolate and a reference strain. Therefore, isolate-specific genetic or biological differences cannot be completely separated from susceptibility-associated features.

Differential expression analysis identified a limited subset of genes that responded to amphotericin B exposure. In *M*. *sympodialis*, a total of 27 Differentially Expressed Genes (DEGs) were identified including 4 upregulated and 23 downregulated genes in resistant relative to susceptible isolates (Figure 1B). In *M*. *furfur*, a total of 82 DEGs were identified, comprising 22 upregulated and 60 downregulated genes (Figure 1D). Thus, amphotericin B exposure was associated with limited gene-specific transcriptional changes superimposed on an otherwise highly similar global transcriptomic profile. Although variability was observed among resistant isolates, samples remained organized by susceptibility phenotype. Differential expression analyses supported this pattern, identifying few DEGs relative to the total expressed in each species. This suggests that amphotericin B resistance involves targeted regulatory adjustments affecting specific cellular functions rather than extensive transcriptome-wide remodeling.^17,23^

To further characterize the biological functions represented among the DEGs, exploratory functional analysis and protein interaction network analyses were performed for each species (Figure 2). In *M*. *sympodialis*, the most represented categories included base excision repair, citrate cycle, transcription, 2-oxocarboxylic acid metabolism, nucleotide excision repair and cell cycle (Figure 2A). Although the cell cycle category showed a nominal *P* value of 0.0486, its FDR was 1.00, and none of the reported categories reached the predefined significance threshold of FDR < 0.05 (Supporting Table S3). We therefore considered these results exploratory and did not interpret them as evidence of statistically significant pathway enrichment. The corresponding protein interaction analysis identified YNK1 as the most connected node in the inferred network (Figure 2B). YNK1 is a particularly relevant node because its function as a nucleoside diphosphate kinase ensures the availability of NTPs and dNTPs that are necessary for DNA transcription and repair. YNK1 showed inferred associations with proteins involved in energy metabolism (IDH2), redox and oxidative-stress responses (TRX2), ribosome-associated functions (RPL18B and RPL4B), transcription-related processes (SPT16 and RPO21), DNA repair (RAD16), and membrane or lipid-related functions (PEX4 and HXT11). As many antifungals induce oxidative damage and cellular stress, this integration could facilitate the DNA repair and cellular adaptation processes associated with antifungal tolerance.^24,25^

**Figure 2:**
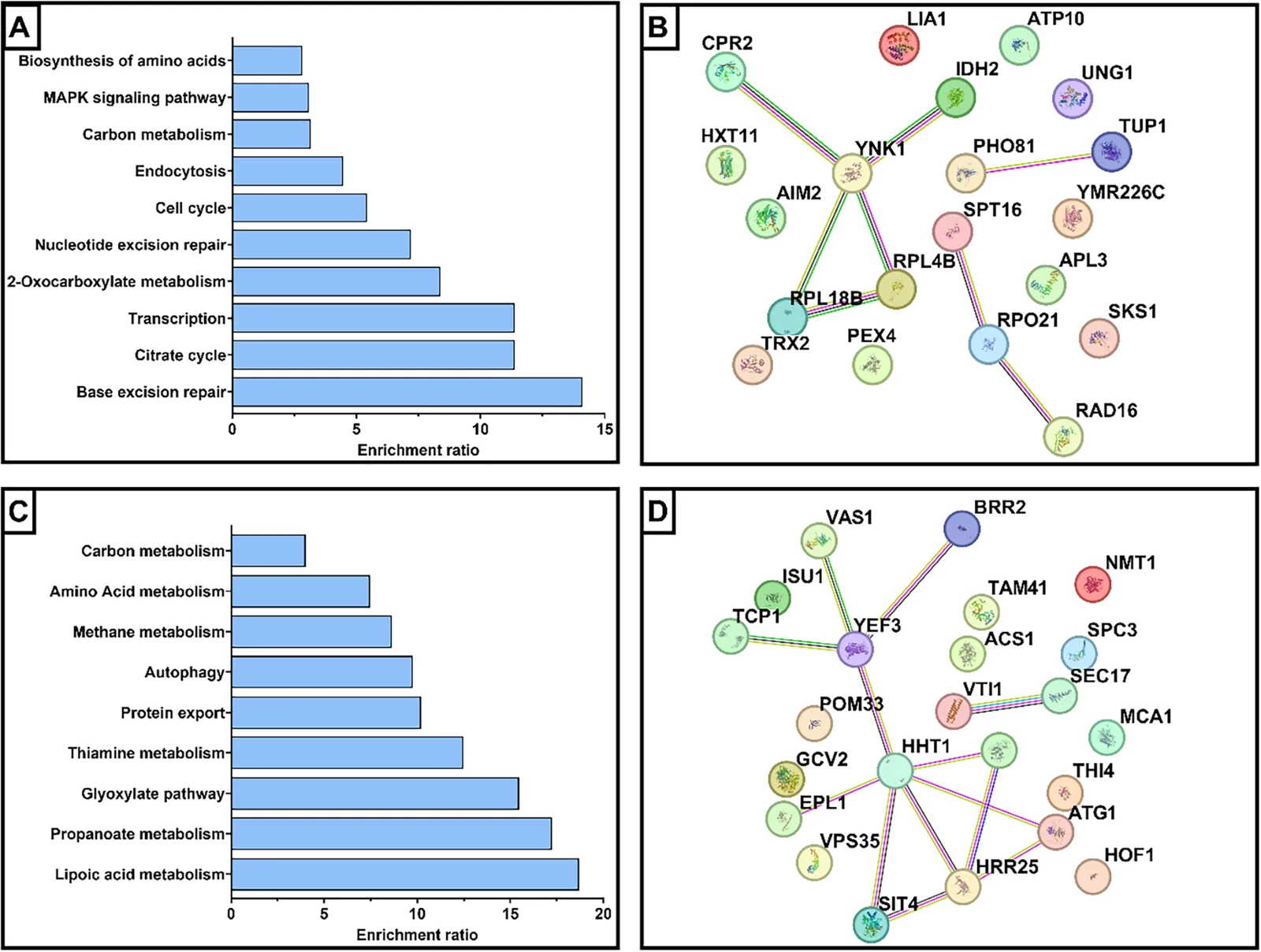
(A) Exploratory functional analysis of biological processes represented among the DEGs in the susceptible *M*. *sympodialis* isolate compared with the resistant isolate. (B) Inferred protein–protein interaction network based on downregulated DEGs in the susceptible *M*. *sympodialis* isolate compared with the resistant isolate. YNK1: nucleoside diphosphate kinase; CPR2, AIM2, and TRX2 are related to oxidative stress, protein folding, and redox regulation; HXT11 is a hexose transporter; RPL18B and RPL4B are ribosomal proteins; PEX4 is involved in peroxisome-associated and lipid-related processes; IDH2 is involved in energy metabolism and the citric acid cycle; LIA1 is involved in protein maturation. (C) Exploratory functional analysis of biological processes represented among the DEGs in the susceptible *M*. *furfur* isolate compared with the resistant isolate. (D) Inferred protein–protein interaction network based on downregulated DEGs in susceptible *M*. *furfur* isolates compared with resistant isolates. YEF3 corresponds to elongation factor 3; HHT1 encodes histone H3; EPL1 is associated with chromatin-remodeling complexes; VTI1 and SEC17 are involved in vesicular trafficking; HRR25 and SIT4 are kinases/phosphatases.

In *M*. *furfur*, lipoic acid metabolism, propanoate metabolism, glyoxylate and dicarboxylate metabolism, thiamine metabolism, protein export, and autophagy were among the most represented categories in the exploratory analysis (Figure 2C). Glyoxylate and dicarboxylate metabolism showed a nominal *P* value of 0.00687; however, its FDR was 0.76984. The remaining reported categories had FDR values of 1.00. Thus, none of the categories met the predefined significance threshold of FDR < 0.05 (Supporting Table S3). The corresponding inferred interaction network contained YEF3 and HHT1 as highly connected nodes (Figure 2D). YEF3 is connected to TCP1, VAS1, BRR2, and HHT1, suggesting links among translation, mitochondrial biogenesis, energy metabolism, RNA processing, and nuclear organization, indicating a strong coupling between protein synthesis and cellular homeostasis. The interactions between HHT1 and HRR25, SIT4, KIN28, and ATG1, among others, point to epigenetic regulation, phosphorylation signaling, cell cycle, and autophagy.^26^

Taken together, functional and interaction network analyses suggest that the transcriptional differences observed between susceptible and resistant *Malassezia* isolates are not enriched in canonical ergosterol biosynthesis genes. The exploratory functional analysis identified several biological processes among the DEGs, including metabolism, stress responses, protein synthesis, and genome maintenance. This transcriptional pattern suggests that resistance is associated with global cellular adaptation mechanisms rather than direct modulation of the primary molecular target of amphotericin B. However, because the pathway-level associations did not remain significant after multiple-testing correction and the protein interaction networks were inferred through orthology to *S*. *cerevisiae*, these analyses should be considered hypothesis-generating rather than evidence of established amphotericin B-resistance mechanisms.

The biological functions associated with these genes may provide hypotheses for future investigation. In particular, the representation of genes related to nucleotide metabolism, redox homeostasis, translation, and DNA repair is compatible with the possibility that broader cellular processes contribute to phenotypic differences between susceptible and resistant isolates. Nevertheless, these interpretations remain provisional because the pathway-level associations did not survive multiple-testing correction and the interaction networks were inferred from an orthologous model system. Thus, the functional and network analyses in the present study should be regarded as hypothesis-generating. They suggest candidate cellular processes that may warrant further investigation but do not establish specific metabolic, redox, translational, or genome-maintenance pathways as mechanisms of amphotericin B resistance.

### Lipidomic Analysis

To assess global lipidomic differences associated with amphotericin B susceptibility, we generated heat maps comparing susceptible and resistant isolates. Because CBS 7222 and CBS 1878 exhibited resistant phenotypes, they were included as resistant reference strains in the exploratory comparisons within their respective species. These strains were not considered biological replicates of the resistant clinical isolates. Instead, their inclusion was intended to provide an additional reference for examining whether some molecular features were shared across strains with the same susceptibility phenotype. Given the limited number of isolates, these comparisons cannot fully separate phenotype-associated features from strain-specific differences.

Approximately 200 lipid molecular species were identified, including FA, glycerolipids, glycerophospholipids, sphingolipids, and sterols. Hierarchical clustering analysis of lipidomic profiles (Figures 3 and 4) revealed differences in the relative abundance profiles of the selected lipid features among the analyzed strains. The observed patterns reflected both susceptibility-group membership and strain origin; however, because reference strains and clinical isolates were not independently replicated, the clustering results cannot establish whether these patterns are specifically attributable to amphotericin B resistance.

**Figure 3:**
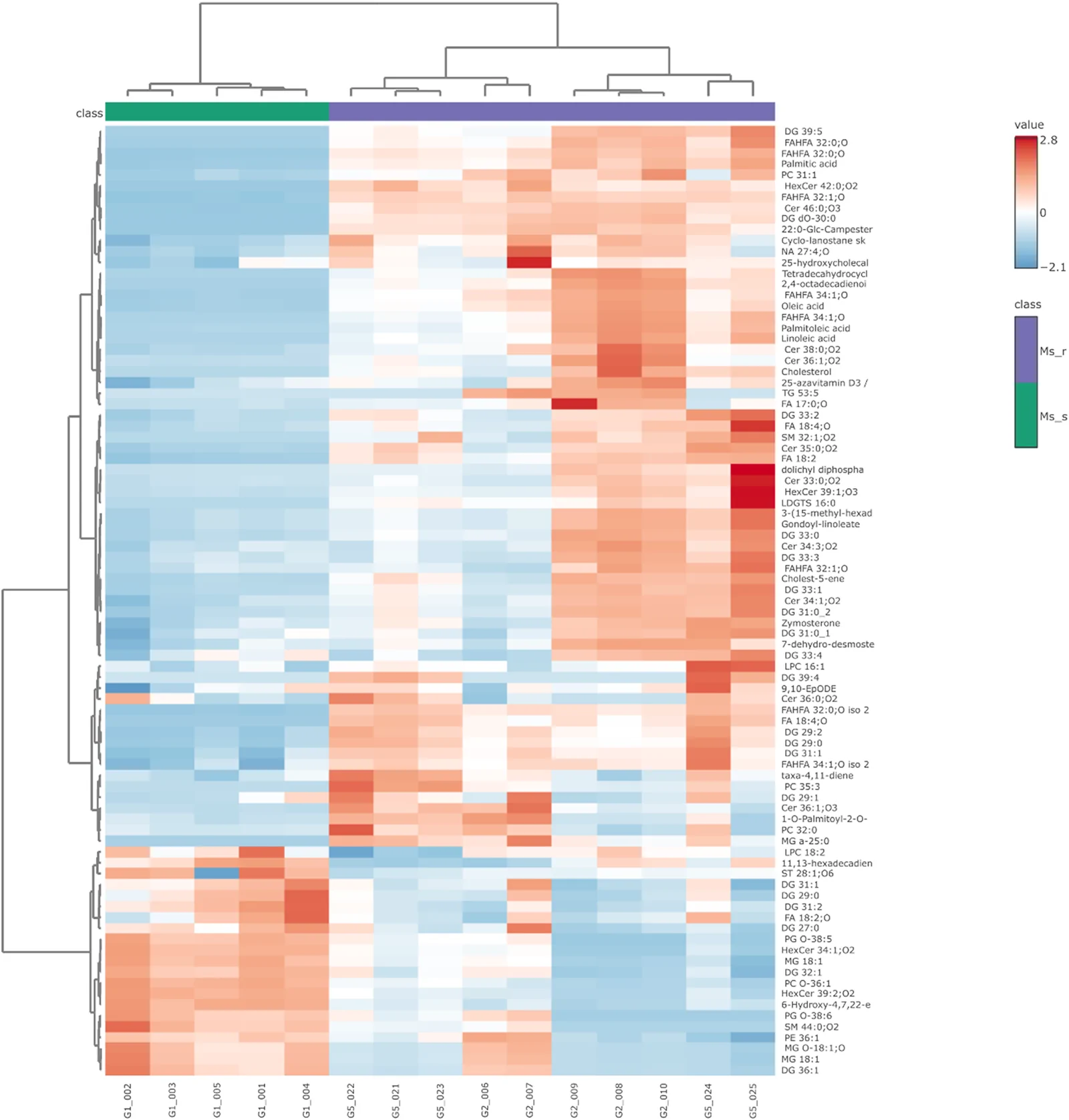
Hierarchical clustering heat map of total lipid abundance in *M*. *sympodialis* susceptible and resistant groups. The heat map displays lipid features filtered by univariate significance (*p* < 0.05; FDR < 0.05) and multivariate importance (VIP ≥ 1.0) derived from OPLS-DA. Data were normalized and scaled using Pareto scaling prior to analysis. Unsupervised hierarchical clustering was performed using Average Linkage algorithm in MetaboAnalyst 6.0. Group annotations: G1/Ms_s (susceptible clinical isolate 20PV, *n* = 5), G2/Ms_r (resistant clinical isolate 1DA, *n* = 5), and G5/Ms_CBS (resistant reference strain CBS 7222, *n* = 5). Rows represent individual lipid features, and columns represent biological replicates. Color intensity indicates relative abundance (standardized Z-scores).

**Figure 4:**
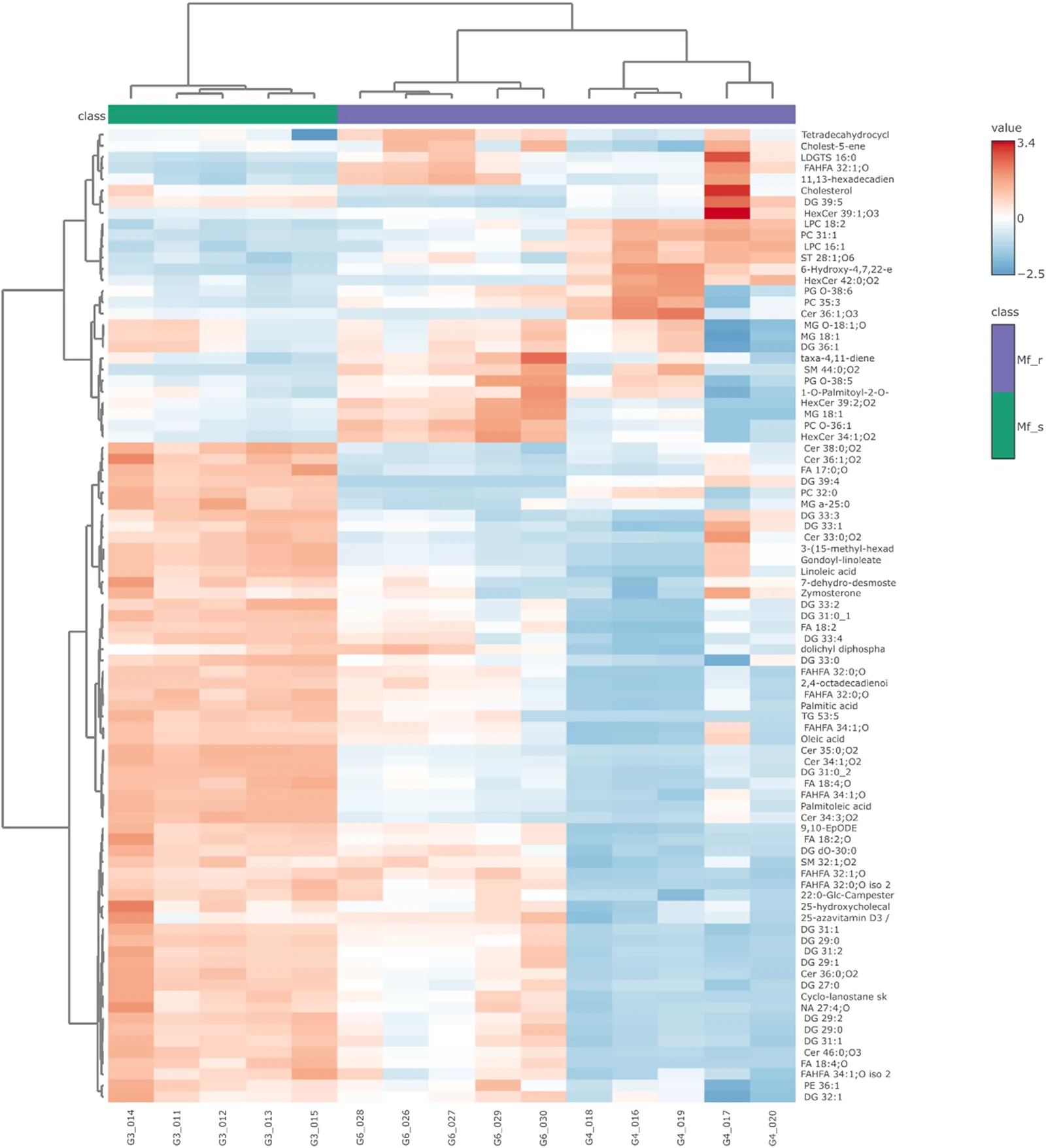
Hierarchical clustering heat map of total lipid abundance in *M*. *furfur* susceptible and resistant groups. The heat map displays lipid features filtered by univariate significance (*p* < 0.05; FDR < 0.05) and multivariate importance (VIP ≥ 1.0) derived from OPLS-DA. Data were normalized and scaled using Pareto scaling prior to analysis. Unsupervised hierarchical clustering was performed using Average Linkage algorithm in MetaboAnalyst 6.0. Group annotations: G3/Mf_s (susceptible clinical isolate 3DA, *n* = 5), G4/Mf_r (resistant clinical isolate 14DS HIV, *n* = 5), and G6/Mf_CBS (resistant reference strain CBS 1878, *n* = 5). Rows represent individual lipid features, and columns represent biological replicates. Color intensity indicates relative abundance (standardized *Z*-scores).

Quantitative screening identified 55 Differential Lipid Molecules (DLMs) out of 91 detected features in *M*. *sympodialis*, and 63 DLMs out of 78 evaluated features in *M*. *furfur*, meeting the criteria of VIP ≥ 1.0 (OPLS-DA) and FDR-adjusted *p* < 0.05. Although aggregate lipid class abundances remained unchanged between groups (Figure S2), species- and strain-specific remodeling was evidenced by significant quantitative shifts in individual lipid molecular species (Supporting Table S4). DLMs were grouped by lipid class with their relative abundance (Supporting Table S5). Therefore, the lipidomic analysis provides evidence that amphotericin B resistance in *Malassezia* is associated with selective changes in individual lipid molecular species rather than with a global alteration of major lipid classes. This distinction is important because the absence of significant differences at the lipid-class level does not preclude biologically relevant remodeling of individual molecular species.

Individual lipid species within these classes showed differential abundance between susceptibility phenotypes. These species-level comparisons are indicated in Figure 5. Regarding sterols, Figure 5A shows a higher relative abundance of cholesterol in the resistant *M*. *sympodialis* isolate. This observation should be interpreted as a difference in the relative abundance of an annotated sterol feature rather than as evidence of global sterol remodeling. Traditionally, ergosterol has been considered the principal fungal sterol. However, other sterols, including lanosterol, brassicasterol, 24-ethylcholesterol, 24-methylcholesterol, and cholesterol, have been shown to characterize different fungal groups.^27^ For example, *Pneumocystis jirovecii*, an important human pathogen, is distinguished by the presence of cholesterol, which has been proposed to contribute to membrane flexibility in its trophic form.^28^ Our lipidomic findings should be considered hypothesis-generating evidence of altered lipid composition rather than definitive evidence that membrane lipid remodeling, independently of sterol remodeling, is the mechanism underlying resistance. Targeted quantitative sterol profiling, including ergosterol and relevant biosynthetic intermediates, using authentic standards and compound-specific calibration, together with functional studies of membrane composition and amphotericin B-membrane interactions, will be necessary to determine the contribution of sterol and nonsterol lipid alterations to resistance.

**Figure 5:**
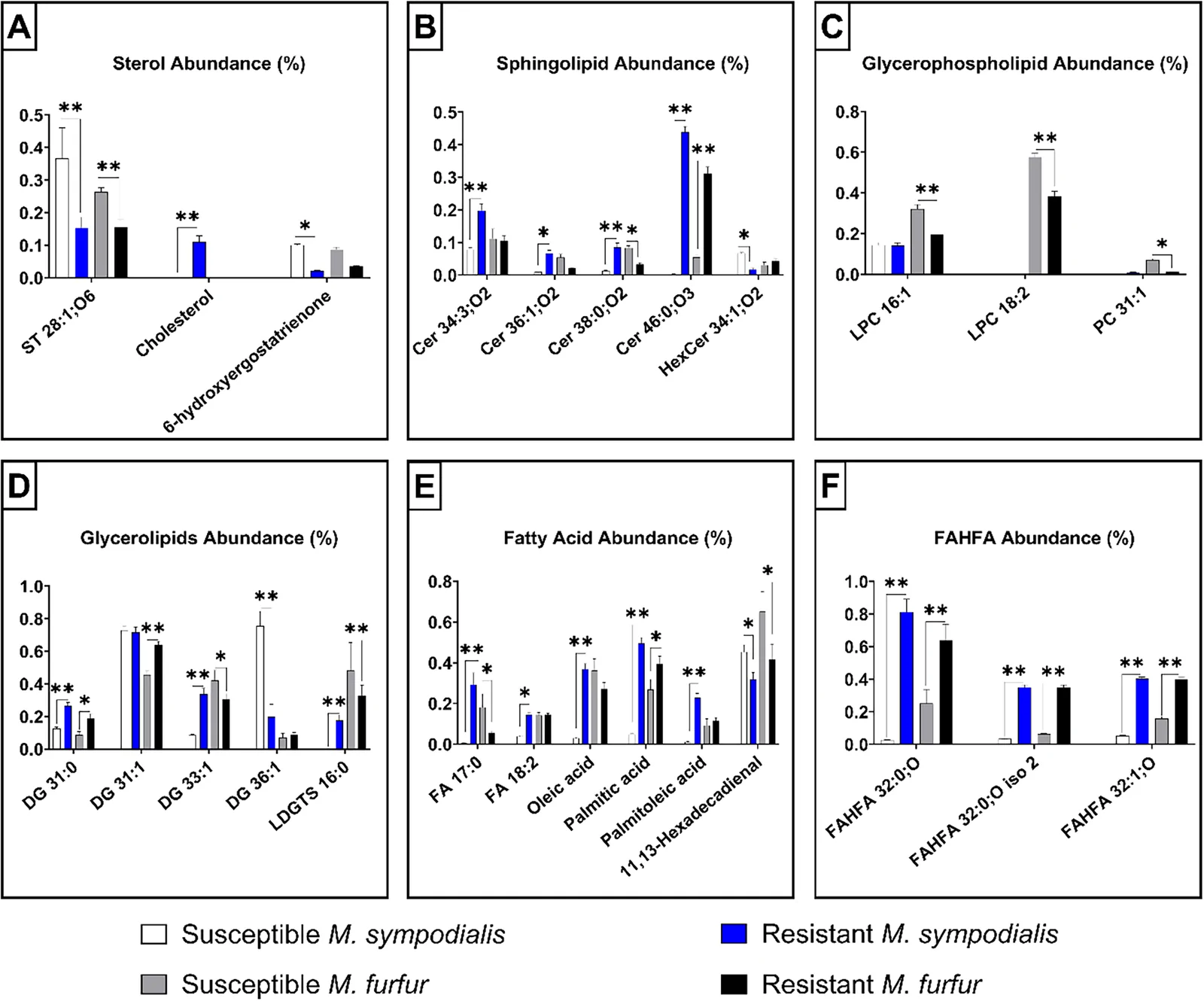
Relative abundance of selected lipid molecular species in *Malassezia* spp. isolates susceptible and resistant to amphotericin B. (A) Sterols. (B) Sphingolipids. (C) Glycerophospholipids. (D) Glycerolipids. (E) Fatty acids. (F) Fatty acyl esters of hydroxy fatty acids (FAHFA). Data are presented as mean ± SD. Statistical significance was assessed using Student’s *t*-test, with FDR correction applied for multiple comparisons. **p < 0.05; *p < 0.01. The complete fold-change, VIP, nominal *p*-value, and FDR-adjusted *p*-value for the analyzed lipid features are provided in Supporting Table S4.

Although amphotericin B exerts its antifungal activity primarily through binding to ergosterol in the fungal membrane, leading to membrane destabilization, pore formation, ion leakage, and ultimately cell death,^29^ no evidence of a marked accumulation of alternative sterol species was observed in resistant isolates. Nevertheless, given the nontargeted nature of the lipidomic workflow, these results should not be interpreted as definitive proof of the absence of sterol remodeling. Targeted sterols analyses, including quantitative determination of ergosterol and its biosynthetic intermediates, would be required to conclusively assess the contribution of sterol composition to amphotericin B resistance in *Malassezia* spp.

In susceptible isolates, ST 28:1;O6 was more abundant in both species, and 6-hydroxy-ergostatrienone showed a higher relative abundance in the susceptible *M*. *sympodialis* isolate. However, because the analysis was not specifically designed for comprehensive sterol profiling or absolute sterol quantification, the absence of prominent differences among the detected sterol features cannot exclude changes in ergosterol abundance, sterol intermediates, or other sterol species that may not have been detected or confidently annotated. Therefore, the observed lipidomic differences are compatible with changes in membrane lipid composition, but their contribution to amphotericin B resistance and their relationship with sterol homeostasis remain unresolved.

Figure 5B shows the relative abundance of sphingolipids. In *M*. *sympodialis*, most molecular species belonging to this lipid class were more abundant in the resistant isolate, including Cer 34:3;O2, Cer 36:1;O2, Cer 38:0;O2, and Cer 46:0;O3, whereas hexosylceramide (HexCer) 34:1;O2 was more abundant in the susceptible isolate. In *M*. *furfur*, the resistant isolate showed a higher relative abundance of Cer 46:0;O3, whereas Cer 38:0;O2 was more abundant in the susceptible isolate. Regarding glycerophospholipids, Figure 5C shows that none exhibited higher relative abundance in resistant isolates of either species. In contrast, higher abundances of lysophosphatidylcholine (LPC) 16:1, LPC 18:2, and PC 31:1 were observed in the susceptible *M*. *furfur* isolate. These findings are consistent with reports on resistant *Candida* spp.^17,30^ and underscore a strong relationship between the homeostasis of this lipid class and drug resistance.^31^ Moreover, sphingolipids significantly influence the growth, virulence, and pathogenic potential of microbial pathogens, including fungi.^32^ Additionally, it has been observed that sterols and sphingolipids interact dynamically, whereby elevated ceramide levels may increase bilayer packing density and promote the reorganization of sterol-containing domains, a mechanism that has been proposed to influence sterol organization and potentially alter amphotericin B accessibility;^33^ however, this possibility was not directly assessed in the present study.

Figure 5D shows the relative abundance of glycerolipids. In *M*. *sympodialis*, a higher abundance of DG 31:0 and DG 33:1 was observed in the resistant isolate, together with increased levels of the betaine lipid lyso-diacylglycerol-trimethyl-homoserine (LDGTS) 16:0, whereas DG 36:1 was more abundant in the susceptible isolate. In *M*. *furfur*, DG 31:0 and DG 31:1 were more abundant in the resistant isolate, while DG 33:1 and LDGTS 16:0 showed higher relative abundance in the susceptible isolate.

Among FA, several molecular species showed significant differences between susceptible and resistant isolates. In *M*. *sympodialis*, palmitic acid, palmitoleic acid, oleic acid, FA 17:0, and FA 18:2 were enriched in resistant isolates, whereas 11,13-hexadecadienal was more abundant in the susceptible isolate (Figure 5E). These differences remained significant after FDR correction (Supporting Table S4). In *M*. *furfur*, palmitic acid was significantly enriched in resistant isolates, while FA 17:0 and 11,13-hexadecadienal were more abundant in susceptible isolates, with the corresponding statistical parameters provided in Supporting Table S4. Importantly, these differences were observed at the level of individual FA species rather than reflecting a significant overall change in the FA class. Collectively, the enrichment of long-chain and predominantly saturated or monounsaturated FA in resistant isolates is consistent with increased membrane order and rigidity, a biophysical state that may limit amphotericin B insertion and pore formation, compatible with a resistance phenotype associated with altered membrane architecture rather than changes in sterol identity.^34^ Our results in *M*. *sympodialis* differ from those reported by Shahi et al., who observed a higher abundance of lipids containing one to three double bonds in their fatty acyl chains in a resistant isolate of *C*. *auris* and proposed that an increased unsaturation index may predict alterations in the membrane lipid environment.^17^ FA are central to fungal physiology and pathogenicity, as well as to their structural role in membranes. They also function as energy substrates through β-oxidation, supporting mitochondrial and peroxisomal metabolism.^35^ In this context, the findings of the present study are consistent with previous reports,^19^ which identified FA with chain lengths ranging from 11 to 28 carbons (including both even- and odd-numbered chains) in six *Malassezia* isolates, predominantly in *M*. *furfur*. Importantly, *Malassezia* species lack complete *de novo* fatty acid synthesis pathways and depend on exogenous lipid sources.^18^ Consequently, selective assimilation and remodeling of medium-derived lipids may contribute to the resistant lipidomic profile observed in this study.

With respect to fatty acyl esters of hydroxy fatty acid (FAHFA), Figure 5F shows a greater relative abundance of FAHFA 32:0;O, FAHFA 32:0;O iso 2, and FAHFA 32:1;O in resistant isolates of both species compared to susceptible ones. FAHFA are a class of complex lipids generated by the esterification of hydroxy FA with fatty acyl chains, representing the convergence of FA biosynthesis, hydroxylation, and acyltransferase-mediated esterification processes. These molecules have attracted growing interest because they may participate in membrane organization, cellular signaling, and stress-response pathways, although their biological roles in fungi remain largely unexplored.^36^

The lipidomic data provide evidence of differences in the relative abundance of individual lipid species between the susceptible and resistant isolates, particularly among ceramides, diacylglycerols, long-chain FA, and FAHFAs. However, these findings should be interpreted as candidate associations rather than definitive mechanisms of amphotericin B resistance. Nevertheless, the limited number of independent isolates and the potential influence of isolate-specific background restrict the extent to which these lipid alterations can be considered generalizable resistance determinants. Validation in larger collections of genetically diverse clinical isolates and targeted quantitative lipidomic analyses will be required to establish whether the identified FA, sphingolipids, glycerolipids, and other lipid species are consistently associated with amphotericin B resistance. Therefore, the lipidomic results are presented as strain- and group-associated differences that require validation in larger collections of clinical isolates.

Additionally, the results of this study suggest differences in lipidomic profiles among *Malassezia* species, independent of antifungal susceptibility, consistent with previous reports.^37^ This pattern is consistent with the differences observed in this investigation when comparing isolates of *M*. *furfur* and *M*. *sympodialis*, reinforcing the hypothesis that species-specific lipidomic profiles exist within the genus.^19^

### Relationship between Transcriptomic and Lipidomic Analysis

PCA was performed by integrating transcriptomic and lipidomic data to assess whether amphotericin B-susceptible and resistant isolates were well separated, which could indicate a combination of genes and lipids associated with susceptibility or resistance to this antifungal. Furthermore, this integrative approach aimed to determine whether resistance is accompanied by a coordinated reorganization of metabolic pathways, gene regulation, and membrane composition. Figure 6A,B show the separation between amphotericin B-susceptible and resistant isolates in each species studied.

**Figure 6:**
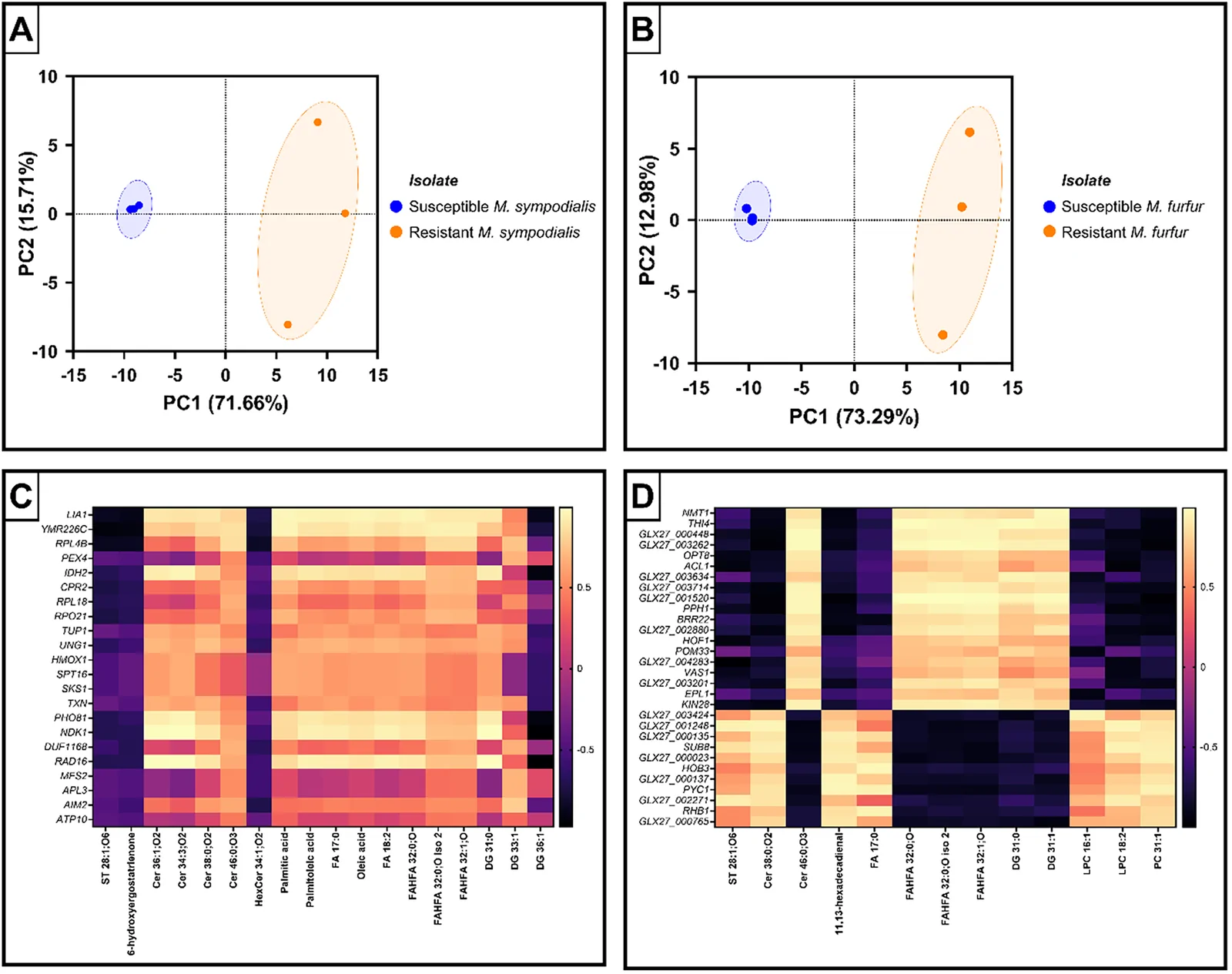
(A) PCA integrating DEGs and statistically significant lipids in *M*. *sympodialis* susceptible and resistant to amphotericin B. (B) PCA integrating DEGs and statistically significant lipids in *M*. *furfur* susceptible and resistant to amphotericin B. (C) Correlation analysis between DEGs and lipids of *M*. *sympodialis*. (D) Correlation analysis between DEGs and lipids of *M*. *furfur*. Yellow indicates a positive correlation, and black indicates a negative correlation.

Finally, Pearson correlation tests were performed, and heat maps were constructed to assess the relationships between DEGs and DLMs for each species. Figure 6C shows 22 genes strongly correlated with 18 lipids for *M*. *sympodialis* (*p* < 0.01), and Figure 6D shows 30 genes strongly correlated with 13 lipids in *M*. *furfur* (*p* < 0.01).

In *M*. *sympodialis*, no repressed genes were found in the resistant isolate that were strongly associated with more than one lipid type. Conversely, overexpressed genes were identified in the resistant isolate, including *LIA1*, *YMR226C*, *IDH2*, *PHO81*, *NDK1*, and *RAD16* (Figure 6C). The genes repressed in the resistant *M*. *furfur* isolate with better annotation and a strong association with multiple lipids were *SUB8* and *PYC1*, while those overexpressed in the resistant isolate were *NMT1*, *THI4*, *OPT8*, *ACL1*, *PPH1*, and *KIN28* (Figure 6D).

Overall, these genes can be grouped into three major functional clusters associated with membrane reorganization, metabolic balance, and stress response. These clusters may partially overlap, suggesting coordinated regulation between the metabolic state, membrane composition, and stress mitigation, which together could contribute to the development of amphotericin B resistance.

In the first cluster, which is related to remodeling and membrane traffic, the genes *SUB8*, *NMT1*, and *KIN28* are included. *SUB8* encodes a subtilisin-like protease (serine protease) that is homologous to the S8A family and is involved in protein degradation and the maturation of secreted or cell surface enzymes. Alterations in surface proteases may influence cell wall integrity and membrane composition, potentially modulating amphotericin B accessibility to ergosterol.^23,38^ In addition, *NMT1* is crucial for N-myristoylation of proteins, which influences membrane trafficking and organization, as well as cell wall biogenesis in fungi. Alterations in *NMT1* could alter the distribution of ergosterol in the membrane, thereby indirectly modulating the efficacy of amphotericin B.^39,40^ *KIN28* encodes transcription factor IIH (TFIIH) kinase. By regulating transcriptional initiation, this regulator can modulate the expression levels of *ERG* genes and efflux pumps, two axes widely implicated in antifungal resistance mechanisms.^41,42^

A second cluster included metabolic regulators linked to acetyl-CoA production and central carbon metabolism. This cluster included the genes *PYC1*, *ACL1*, *YMR226C*, *IDH2*, and *NDK1*. Because acetyl-CoA is a key precursor for FA and sterol biosynthesis and degradation, modulation of its availability may influence membrane lipid composition and ergosterol organization.^43^ In this context, resistance may be supported by metabolic flux redistribution that stabilizes the altered lipid profile observed in resistant isolates.^44^ *PYC1* encodes one of the two pyruvate carboxylase isoenzymes, which catalyzes the conversion of pyruvate to oxaloacetate. This enzyme participates in gluconeogenesis but is also linked to the tricarboxylic acid cycle, thereby contributing to metabolic balance by regulating anaplerotic reactions and indirectly supplying precursors for acetyl-CoA generation and ergosterol synthesis.^45^

Another gene involved in central metabolism is *IDH2*, which encodes a mitochondrial isocitrate dehydrogenase. This enzyme plays a crucial role in the tricarboxylic acid cycle, aiding in the production of metabolic intermediates and reducing the number of equivalents required for biosynthetic pathways, such as lipogenesis. Consequently, alterations in its activity could influence lipid synthesis and pathways that supply precursors for ergosterol biosynthesis, thereby potentially affecting membrane composition. However, direct evidence linking *IDH2* to antifungal resistance remains limited, and this potential contribution should therefore be considered physiologically plausible but largely inferred from its metabolic role.^46^

In parallel, *ACL1* encodes a subunit of ATP-citrate lyase, a key enzyme for the generation of cytosolic acetyl-CoA. In *C*. *neoformans*, *ACL1*-deficient mutants exhibit reduced acetyl-CoA levels, slower growth on glucose, defects in virulence factors, and increased susceptibility to fluconazole, highlighting the importance of *ACL1* in lipid homeostasis and in responses to antifungals that affect ergosterol biosynthesis. While there is no direct evidence linking *ACL1* to resistance to amphotericin B, its central role in supplying lipid precursors suggests that it may indirectly influence susceptibility to this antifungal.^47^

The other two genes associated with the second cluster were *YMR226C* and *NDK1*. *YMR226C* encodes a short-chain dehydrogenase/reductase in *S*. *cerevisiae* that may influence the cellular availability of NADPH, an essential cofactor for FA and sterol biosynthesis.^48,49^ In contrast, *NDK1* encodes a nucleoside diphosphate kinase that catalyzes phosphate exchange between nucleotides. By maintaining balanced NTP/NDP pools, this enzyme plays an important role in sustaining cellular metabolic homeostasis.^50^

The third cluster comprises *PPH1*, *LIA1*, *THI4*, *OPT8*, *RAD16*, and *PHO81*, genes associated with cellular stress tolerance. *PPH1* encodes phosphatase type 2A (PP2A), an enzyme that regulates stress response and TOR (Target of Rapamycin) signaling pathways.^51^ *LIA1* encodes deoxyhypusine monooxygenase, an enzyme involved in the post-translational modification of eukaryotic translation initiation factor 5A (eIF5A) and plays a role in cytoskeleton organization in yeast. Although no studies have directly linked it to antifungal resistance, its roles in translation and cellular homeostasis suggest it could modulate general stress responses.^52^ Additionally, *THI4* is involved in thiamine biosynthesis, which increases tolerance to oxidative, osmotic, and thermal stress, states that often accompany antifungal exposure.^44^ *OPT8* encodes an oligopeptide transporter, and although there is no direct evidence linking it to amphotericin B resistance, its function suggests a possible indirect role in the stress response.^53^

*RAD16* is a component of nucleotide excision repair pathway and may contribute to increased capacity for DNA repair and response to damage, particularly to antifungal-induced oxidative stress.^54^ Finally, *PHO81* encodes an inhibitor of the cyclin-CDK (Cyclin-Dependent Kinase) complex in the phosphate signaling pathway of pathogenic fungi. Several studies have reported that this gene is linked to phosphate-starvation-induced pexophagy.^55^ Pexophagy represents a selective form of autophagy in which peroxisomes are degraded in response to oxidative stress, nutrient limitation, and imbalances in long-chain FA metabolism.^56^

## Conclusions

The present study identified transcriptomic and lipidomic differences among the *Malassezia* strains analyzed according to their amphotericin B susceptibility phenotype and strain origin. The results suggest that amphotericin B resistance in *Malassezia* may not primarily depend on direct alterations in ergosterol biosynthesis but rather on coordinated metabolic and regulatory adaptations that favor cellular adaptation to antifungal stress. Integration of transcriptomic and lipidomic analyses revealed that resistant isolates exhibited coordinated changes in gene expression and lipid composition, as evidenced by modulation of central pathways in energy metabolism, redox homeostasis, transcriptional regulation, and lipid remodeling. This pattern was reflected in differences in the relative abundance of selected lipid molecular species across FA, glycerolipids, sphingolipids, and other lipid classes, which could help maintain membrane integrity and function. Taken together, these changes could modify the lipid context of the membrane and potentially attenuate the impact of amphotericin B, suggesting a resistance phenotype associated with systemic metabolic adjustments rather than the modification of a single molecular target.

The findings suggest that metabolic and membrane-associated features may be relevant to the observed differences, but they do not establish a definitive molecular mechanism of resistance. Our results broaden the understanding of the molecular and metabolic mechanisms underlying resistance in *Malassezia* spp. and underscore the importance of integrated multiomics approaches for the early identification of strains with resistance potential, thereby contributing to the design of more precise and effective therapeutic strategies in human and veterinary medicine.

This study has limitations that should be considered when interpreting the findings. First, the number of isolates was limited, with one susceptible and one resistant clinical isolate available for each species, complemented by one resistant reference strain per species. Although CBS 7222 and CBS 1878 displayed resistant phenotypes, they originated from different sources and may differ from the clinical isolates in their genetic background, laboratory history, and adaptation to culture conditions. Consequently, the present design does not allow resistance-associated molecular features to be completely separated from strain-specific effects. The transcriptomic and lipidomic differences identified here should therefore be interpreted as candidate strain- or group-associated features rather than definitive mechanisms of amphotericin B resistance. Larger studies including multiple independent clinical isolates with well-characterized susceptibility phenotypes and matched clinical metadata will be necessary to validate these associations. Second, only *M*. *furfur* and *M*. *sympodialis* were investigated. Because *M*. *pachydermatis* is also an important species associated with invasive infections, the absence of this species limits the extrapolation of the findings to the broader spectrum of invasive *Malassezia* infections. Third, our study lacked direct functional validation, such as gene disruption/overexpression models or biophysical membrane interaction assays, to experimentally confirm the physiological role of the identified candidate genes and lipids, so the integrated transcriptomic-lipidomic associations should be considered hypothesis-generating rather than evidence of causality. Validation using larger collections of genetically diverse clinical isolates, including multiple susceptible and resistant isolates of *Malassezia* spp., as well as targeted lipid and functional analyses, will be necessary to determine the reproducibility and biological relevance of the candidate resistance-associated features identified here.

Future studies should integrate targeted quantitative profiling of ergosterol and biosynthetic sterol intermediates, alongside genetic manipulation tools (e.g., gene disruption or overexpression models) and biophysical membrane interaction assays, to conclusively validate the functional mechanisms underlying amphotericin B resistance in *Malassezia* species.

## Methods

### *Malassezia* spp. Isolates and Susceptibility Profile

Four *Malassezia* spp. isolates were evaluated in this study. These isolates were previously obtained from patients with various skin conditions (as pityriasis versicolor, atopic dermatitis, and seborrheic dermatitis) and are part of the collection of the CeMoP Research Group at Universidad de los Andes.^57^ Two of these isolates were previously identified as *M*. *sympodialis*, and the other two as *M*. *furfur*, using ribosomal gene amplification by conventional PCR, sequencing, and phylogenetic analysis.^58^ Additionally, we included reference strains *M*. *sympodialis* CBS 7222 and *M*. *furfur* CBS 1878 (Westerdijk Institute, Utrecht, The Netherlands) (Table 1). The amphotericin B susceptibility profile was determined for a collection of 26 clinical isolates and two reference strains of *Malassezia* spp. MICs were determined by broth microdilution according to the CLSI M27-A3 procedure with some modifications to meet the nutritional requirements of *Malassezia*.^59,60^ Three replicate MIC determinations were performed for each isolate or reference strain. Since no breakpoints have been defined to categorize *Malassezia* spp. isolates as susceptible or resistant to amphotericin B, the following criteria were used:^21^

1 $$S\mathrm{usceptible Isolate}\;\left(S\right)=\left\{i:\mathrm{MI}C_{i}\leq \mathrm{MI}C_{50}\right\}$$

2 $$\mathrm{Dose}-\mathrm{Dependent Susceptible Isolate}\;\left(\mathrm{DDS}\right)=\left\{i:\mathrm{MI}C_{50}<\mathrm{MI}C_{i}\leq \mathrm{MI}C_{90}\right\}$$

3 $$\mathrm{Resistant Isolate}\;\left(R\right)=\left\{i:\mathrm{MI}C_{i}>\mathrm{MI}C_{90}\right\}$$

These categories were used as operational phenotypic classifications and do not represent validated clinical breakpoints for amphotericin B in *Malassezia* spp.

### Determination of Subinhibitory Concentration of Amphotericin B

Isolates and reference strains were exposed to different concentrations of amphotericin B using the agar dilution “spot” assay.^61^ Briefly, 5-day-old colonies on modified Dixon agar (Mycosel agar [BD, USA] 36 g L^–1^, Oxbile [Sigma-Aldrich, USA] 20 g L^–1^, malt extract [Oxoid, USA] 36 g L^–1^, glycerol [Sigma-Aldrich, USA] 2 mL L^–1^, oleic acid [Sigma-Aldrich, USA] 2 mL L^–1^ and Tween 40 [Sigma-Aldrich, USA] 10 mL L^–1^) were suspended in 5 mL of distilled water with 1% Tween 80 until a visual turbidity of approximately 2.0 McFarland units was reached. Subsequently, 3 mL of this suspension was added to 27 mL of Dixon broth, which was then incubated at 32 °C and 180 rpm for 3 days. After this time, 300 μL of the culture was added to 29.7 mL of Dixon broth, and the mixture was incubated again at 32 °C and 180 rpm for 20–24 h to allow the cells to reach exponential growth. To adjust the inoculum to a concentration of 1 × 10^6^ cells/mL, 100 μL of the culture was added to 900 μL of 1% Tween 80 (concentration 10^–1^), then 100 μL was added to another 900 μL of 1% Tween 80 (concentration 10^–2^) and counted in a Neubauer chamber.^62^ Subsequently, 5 μL of these suspensions were taken and placed on the surface of Petri dishes containing modified Dixon agar in the absence (control) or presence of amphotericin B (Sigma-Aldrich, USA) at concentrations of 0.03–16 μg/mL, which were incubated at 32 °C for 5 days. The subinhibitory concentration of amphotericin B was determined as the highest concentration of the antifungal agent that did not inhibit growth on the agar surface.

### Transcriptomic Analysis

As mentioned above, cell density was assessed by Neubauer chamber counting to standardize the inoculum to 1 × 10^6^ cells/mL. Susceptible and resistant isolates, including reference strains, were cultured under exactly identical conditions, exposed to their corresponding subinhibitory concentration of amphotericin B for 20–24 h, and allow the cells to reach exponential growth, before RNA extraction to induce the transcriptional response associated with antifungal stress as previously described for fungal transcriptomic analyses.^63^ The suspensions were then washed three times with phosphate-buffered saline (PBS) and centrifuged at 1248 g for 10 min. Three replicates were performed for each isolate.

Total RNA extraction was performed using the NucleoSpin RNA kit (Macherey-Nagel, Germany) following the manufacturer’s instructions, with a modification using liquid nitrogen and a pellet pestle attached to a drill to disrupt the yeast cell wall more efficiently. Total RNA concentration was determined spectrophotometrically using NanoDrop (Thermo Fisher Scientific, USA), and its quality was confirmed by electrophoresis on a 1% agarose gel with 1% sodium hypochlorite (The Clorox Company, USA).^64^ mRNA was isolated from total RNA using the NEBNext Poly­(A) mRNA Magnetic Isolation Module (New England Biolabs Inc., USA) and quantified as described above.^65^ Conversion to cDNA was performed using the LunaScript RT SuperMix Kit (New England Biolabs Inc., USA), and the concentration was determined fluorometrically using the Qubit dsDNA HS kit on a FluorQuant (Loccus, Brazil). Before sequencing, the library was prepared, including end repair, using the NEBNext Ultra II End Repair/Da-Tailing Module (New England Biolabs Inc., USA). The cDNA was labeled using Native Barcoding Expansion 96 EXP-NBD196 (Oxford Nanopore Technologies, UK) with the NEBNext Ultra II Ligation Module (New England Biolabs Inc., USA). The cDNA samples were pooled in a 1.5 mL tube and cleaned and purified using AMPure XP magnetic beads (Beckman Colter, USA). Ligation of the AMII adapter and final library preparation were performed using the NEBNext Quick Ligation Module (New England Biolabs Inc., USA) and Ligation Sequencing SQK-LSK109 kits (Oxford Nanopore Technologies, UK). Finally, the library was loaded into the Flow cell R10.4.1 and sequenced using a MinION Mk1C (Oxford Nanopore Technologies, UK).

Transcriptomic sequencing data were subjected to bioinformatic processing and downstream analyses. These analyses were conducted by the Grupo de Genómica de Microorganismos Emergentes of the Research Directorate at the National Institute of Health of Colombia. Raw Nanopore sequencing data were basecalled and demultiplexed using Dorado (v1.1.1) (Oxford Nanopore Technologies, UK). Initial quality assessment of the reads was performed using NanoPlot (v1.46.2). Adapter trimming was conducted with Porechop (v0.2.4), and low-quality and chimeric reads were filtered using NanoFilt (v2.8.0).^66^ Reads that met the quality and length criteria were retained for further analysis. The filtered reads were independently aligned to the reference genomes of *M*. *sympodialis* (ASM34930v2; https://www.ncbi.nlm.nih.gov/datasets/genome/GCF_000349305.1/) and *M*. *furfur* (ASM993813v1; https://www.ncbi.nlm.nih.gov/datasets/genome/GCA_009938135.1/) using Minimap2 (v2.28)^67^ with parameters optimized for long-read transcriptomic data. Gene expression quantification was performed using FeatureCounts from the Rsubread v2.4.3 package^68^ to generate gene-level count matrices for each species. The count data were subsequently analyzed to search for differential gene expression levels and statistical significance using the DESeq2 package (v1.40.2).^69^ Genes with an absolute log2 (fold change) value ≥ 2 or ≤ −2, and an adjusted *p*-value *(padj)* ≤ 0.05, calculated using the Benjamini-Hochberg method, were considered DEGs 86 lipids were identified as DLMs in the comparative analysis. To explore molecular interactions among DEGs, separate interaction networks were constructed for these genes in each species using STRING (v.12.0)^70^ by orthological mapping with *S*. *cerevisiae*, given the limited interaction data for *Malassezia* spp. The *S*. *cerevisiae* reference network was used to infer conserved functional interactions. A confidence score threshold of 0.7 was applied, restricting interaction sources to Experiments, Databases, and Coexpression. Network clustering was performed using the Markov Cluster Algorithm (MCL). Further network visualization and topological analysis were conducted in Cytoscape (v.3.10).^71^ To assess the biological significance of the identified DEGs, a functional enrichment analysis was performed using WebGestalt 2024 (a web-based Gene SeT Analysis Toolkit),^72^ focusing on biological process terms from Gene Ontology (GO; https://geneontology.org/), a structured database that classifies gene functions into categories of biological processes, molecular functions, and cellular components.^73^

### Lipidomic Analysis

The lipid compositions of the isolates and reference strains were determined. Extraction and analysis were performed at the Metabolomics Core Facility (http://corefacilities.uniandes.edu.co/metcore/) of Universidad de los Andes. To do this, aliquots of *Malassezia* spp., in the exponential phase, were washed and centrifuged as mentioned above. Each sample was lyophilized and weighed before analysis. Lipid extraction was performed using a previously described method, with minor modifications.^74^ Briefly, extraction was performed using a biphasic solvent system of cold methanol, methyl tert-butyl ether (MTBE), and water. For every 100 mg of sample, 225 μL of methanol, 750 μL of MTBE, and 188 μL of water were added; each sample was vortexed for 10 s, then all samples were vortexed for 15 min after each solvent addition. All samples were centrifuged for 10 min at 13,000 rpm, and the upper phase was transferred to another tube. Five replicates were performed for each isolate.

The samples were analyzed using an Agilent Technologies 1260 liquid chromatography (LC) system coupled to a 6545 Q-TOF quadrupole mass spectrometer (MS) with electrospray ionization (ESI). Ten microliters of the sample were injected into a C18 column (100 × 3.0 mM, 2.7 μm) at 60 °C and a gradient elution consisting of: 60:40 acetonitrile: water +10 mM ammonium formate +0.1% formic acid (Phase A) and 90:10 isopropyl alcohol: acetonitrile +10 mM ammonium formate +0.1% formic acid (Phase B) with a constant flow rate of 0.6 mL/min. Mass spectrometry detection was performed in positive ESI mode with a *full scan* from 100 to 1700 *m*/*z*. Throughout the analysis, two reference masses were used for mass correction: *m*/*z* 121.0509 (C_5_H_4_N_4_) and *m*/*z* 922.0098 (C_18_H_18_O_6_N_3_P_3_F_24_). Quality control (QC) samples were prepared by mixing equal volumes of extract. To assess the reproducibility of sample preparation and the stability of the analytical platform, several QC runs were performed until the analytical system equilibrated.

Lipidomic profiling was performed using an HPLC-QTOF-MS-based nontargeted workflow. In this approach, lipid molecular features were detected across the analytical LC-MS space without restricting the analysis to a predefined panel of target compounds. Molecular features were subsequently annotated according to the available analytical information and annotation confidence. The workflow therefore provided broad coverage of multiple lipid classes, including fatty acids, glycerolipids, glycerophospholipids, sphingolipids, and sterols, but was not specifically optimized for exhaustive sterol profiling or absolute quantification of ergosterol and its biosynthetic intermediates.

The data were processed with Agilent MassHunter Profinder B.10.0 software for deconvolution, alignment, and integration. Compounds were retained after applying a reproducibility filter based on 100% detection within groups and a QC coefficient of variation below 20%. Univariate and multivariate analyses identified lipid features associated with amphotericin B susceptibility. For univariate analysis, statistical significance was assessed using Student’s *t*-test, followed by false discovery rate (FDR) correction, while OPLS-DA was used as the multivariate approach. To assess the model’s robustness and rule out overfitting, a permutation analysis was performed by comparing the observed values with those from randomly generated models. *Pareto* scaling was used to identify the molecular characteristics responsible for the separation between the groups. DLMs were defined using a nominal *p* < 0.05 and Variable Importance in Projection (VIP) > 1. The complete statistical information, including fold change, VIP, *p*-value, and FDR-adjusted *p*-value, is provided in Supporting Table S4. The annotation of statistically significant molecular characteristics was performed using various tools, including exact mass, molecular formula, mass spectra, and retention times, with MS-DIAL software version 5.1.230912. This software includes an internal database containing theoretical MS/MS spectra for lipid annotations and the comprehensive atlas ″MS-DIAL LipidBlast (version 68)″. Each metabolite was reported with a confidence-level ID in accordance with the guidelines established by the Metabolomics Standards Initiative (MSI). These guidelines define four levels: level 1 for metabolites identified using chemical standards, level 2 for metabolites with spectral match in reference databases, level 3 for putative compounds that have agreement in molecular formula and MS/MS data, and level 4 for metabolites that only match MS1 in online databases.^75^ Lipids were expressed in the format Class X:Y, where X corresponds to the total number of carbons and Y to the number of double bonds present in the acyl chains. In some cases, additional modifiers such as “;n” are included, indicating the number of hydroxyl groups.

### Statistical Analysis

The results are shown as mean ± standard deviation (SD), and experiments were performed in triplicate. Data analysis and graphical representation were generated using GraphPad Prism version 10.5 (GraphPad Software, USA), DESeq2 version 1.40.2,^69^ WebGestalt 2024,^72^ STRING version 12.0,^70^ and MetaboAnalyst 6.0.^76^ Statistical significance was set at *p* < 0.05.

Hierarchical clustering heat maps were constructed in MetaboAnalyst 6.0 using normalized data subjected to Pareto scaling. Features included in the heat maps were restricted to statistically significant lipid features meeting criteria of VIP ≥ 1.0 and nominal *p* < 0.05 (FDR < 0.05). Dendrograms were generated using unsupervised hierarchical clustering using Average Linkage algorithm as the similarity metric. Mappings were annotated by susceptibility phenotype and strain origin across five biological replicates per condition (*n* = 5).

PCA to evaluate relationship between transcriptomic and lipidomic analysis was performed using all DLMs and DEGs identified in the isolates. *K*-means clustering was applied to identify groups associated with amphotericin B resistance profiles. Statistical significance was defined as *p* < 0.05. Pearson correlation analyses were conducted to evaluate associations between DLMs and DEGs related to amphotericin B resistance, and correlation heatmaps were generated in GraphPad Prism version 10.5 using a significance threshold of *p* < 0.01.

## Supplementary Material



## Notes

Figure S1: Correlation plot comparing resistant *Malassezia* spp. with exposure and without exposure to amphotericin B. Figure S2: Relative abundance of lipids grouped by lipid class in amphotericin B-susceptible and resistant *Malassezia* spp. isolates. Table S1: Nanopore sequencing quality metrics before and after preprocessing. Table S2: Alignment statistics of Nanopore transcriptomic reads against the corresponding reference genomes using Minimap2. Table S3: Exploratory functonal enrichment analysis of DEGs in *M. sympodialis* and *M. furfur*. Table S4: Statistical analysis of lipid species in amphotericin B-susceptible and resistant *Malassezia* isolates. Table S5: Relative abundance of DLMs by class when comparing *Malassezia* spp. susceptible and resistant to amphotericin B (PDF)
