# Supplementary material for: Comparative Transcriptomic and Lipidomic Analysis of Amphotericin B‑Susceptible and Resistant Clinical Isolates of Malassezia spp
Source: ACS Omega. 2026 Oct 27;11(44):69193–208. doi: 10.1021/acsomega.6c09344 (PMC13756745; doi:10.1021/acsomega.6c09344)
Supplement: Supplementary file 1 [file ao6c09344_si_001.pdf]

Supporting Information

Comparative transcriptomic and lipidomic analysis of amphotericin B-susceptible and resistant clinical isolates of *Malassezia* spp.

Juan Camilo Galvis-Marín[a], Fredy Alexander Tabares-Villa[a], Augusto Zuluaga-Vélez[a], Paola Rojas-Estevez[b], Mónica P. Cala[c], Adriana Marcela Celis-Ramírez[d*], Juan Carlos Sepúlveda-Arias[a*]

[a] Grupo Infección e Inmunidad, Facultad Ciencias de la Salud, Universidad Tecnológica de Pereira, Carrera 27 #10-02 Barrio Álamos, Pereira, Risaralda, Colombia - Zip code: 660003
[b] Grupo de Investigación Genómica de Microorganismos Emergentes, Dirección de Investigación, Instituto Nacional de Salud, Avenida Calle 26 #51-20, Zona 6 CAN, Bogotá DC, Colombia - Zip code: 111321
[c] Metabolomics Core Facility (MetCore), Vicepresidency for Research, Universidad de los Andes, Cra 1 #18A-12 Bogotá DC, Colombia - Zip code: 111711
[d] Grupo de Investigación Celular y Molecular de Microorganismos Patógenos (CeMoP), Facultad de Ciencias, Universidad de los Andes, Cra 1 #18A-12 Bogotá DC, Colombia - Zip code: 111711

* Corresponding authors:

Juan Carlos Sepúlveda Arias
Email address: jcsepulv@utp.edu.co
Phone: 57 3002071669

Adriana Marcela Celis Ramírez
Email address: acelis@uniandes.edu.co
Phone: 57 601 3394949 ext 3857

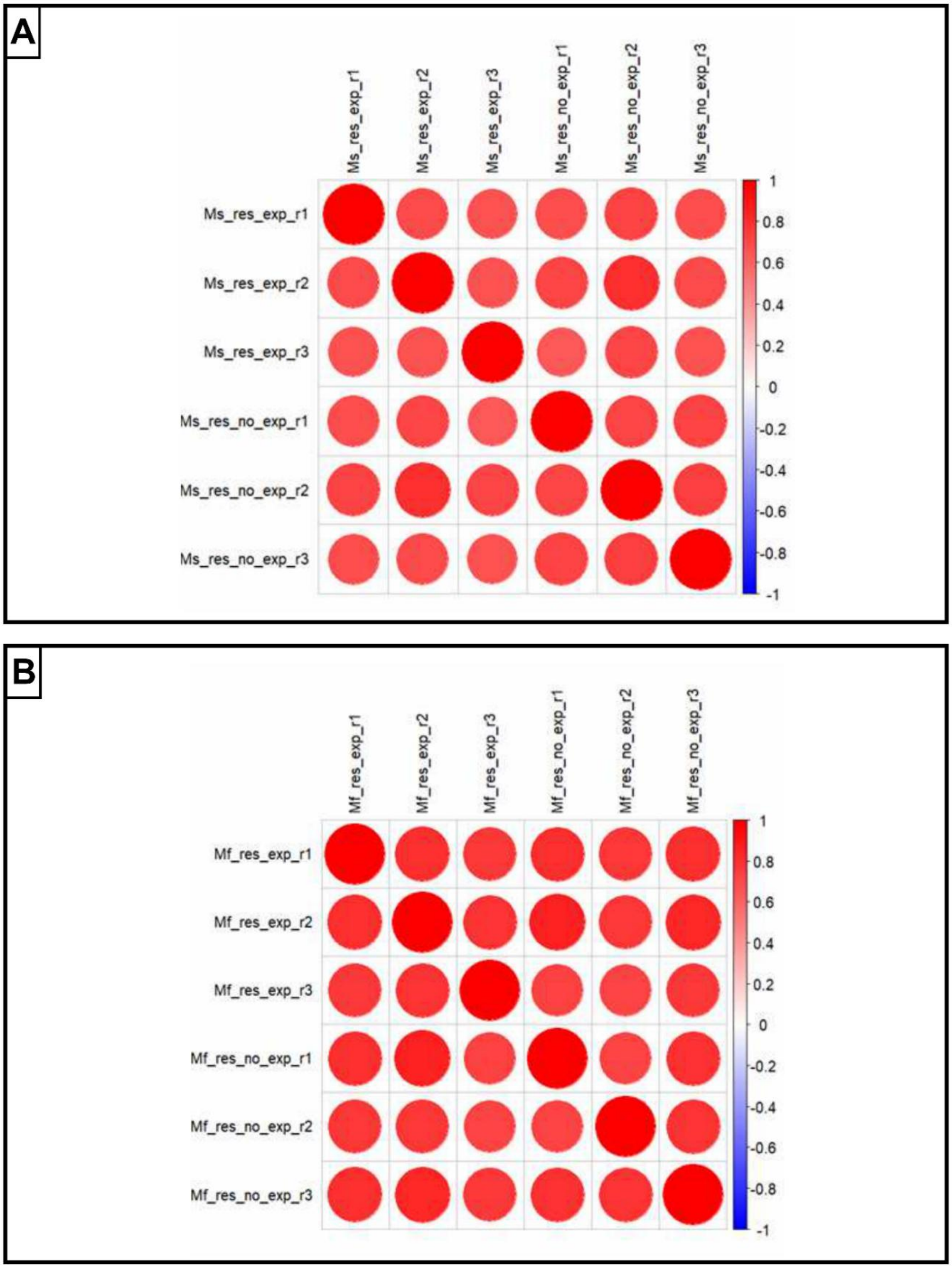

Figure S1. Correlation plot comparing resistant *Malassezia* spp. with exposure and without exposure to amphotericin B. **A.** *M. sympodialis.* **B.** *M. furfur.* Red: positive correlation. Blue: negative correlation. Each circle represents the correlation coefficient between the corresponding pairs of variables. The larger the circle, the greater the magnitude of the correlation coefficient (closer to ±1). Ms_res_exp: *M. sympodialis* with exposure; Ms_res_no_exp: *M. sympodialis* without exposure; Mf_res_exp: *M. furfur* with exposure; Mf_res_no_exp: *M. furfur* without exposure; r1, r2, and r3: experimental replicates.

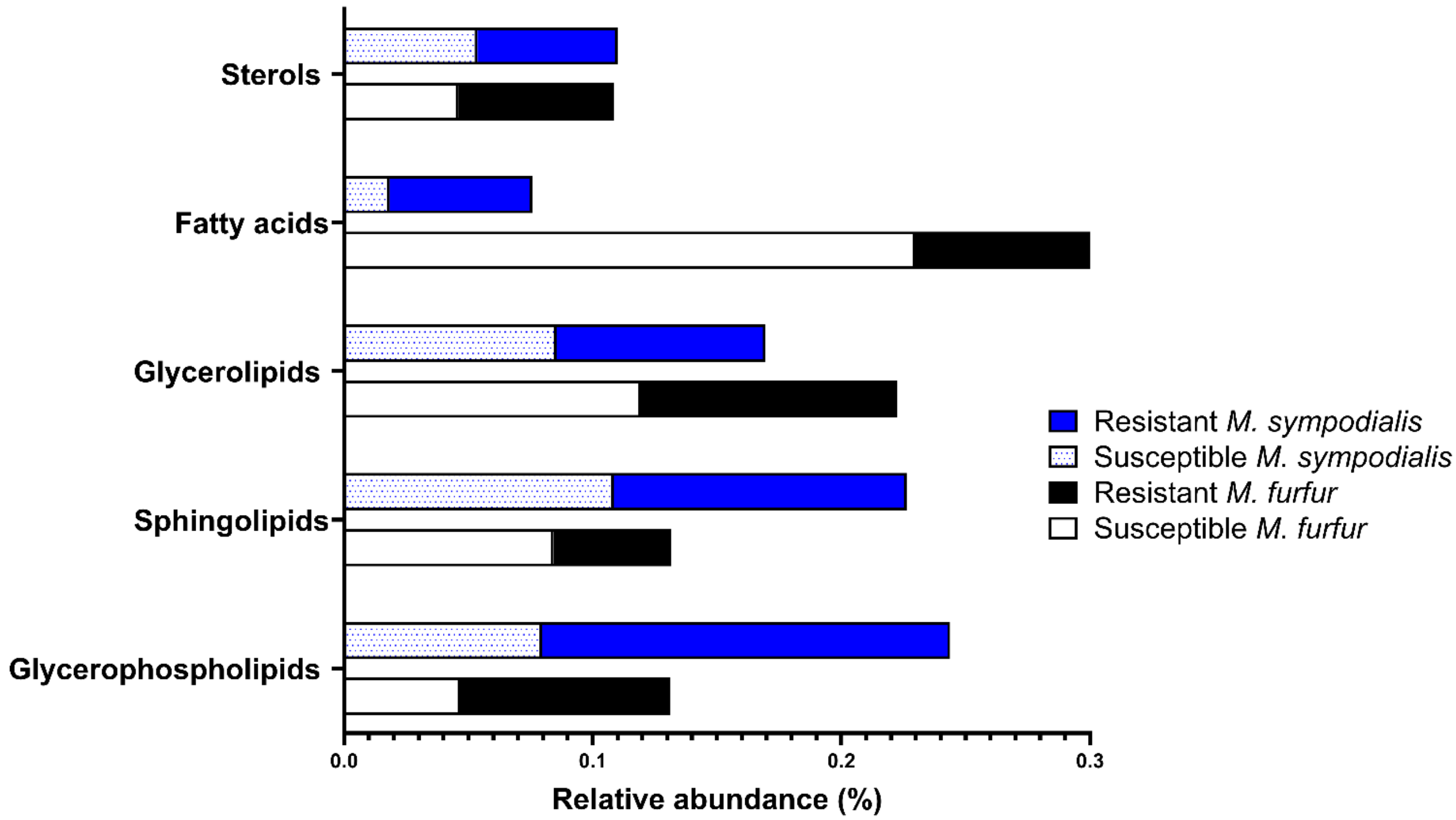

Figure S2. Relative abundance of lipids grouped by lipid class in amphotericin B-susceptible and resistant *Malassezia* spp. isolates.

Table S1. Nanopore sequencing quality metrics before and after preprocessing.

Species	Stage	Reads	Mean length	Median length	Mean Q	N50
M. sympodialis	Before filtering	176686	588.6	475	14.4	661
	After filtering	176622	588.8	475	14.4	661
M. furfur	Before filtering	216211	588.4	478	14.3	666
	After filtering	216163	588.6	478	14.3	666

Table S2. Alignment statistics of Nanopore transcriptomic reads against the corresponding reference genomes using Minimap2.

Species	Categorized	AMB[1] exposure	Replicate	Sample	Total Reads	Mapped Reads	% Mapped	Avg. % Mapped
M. sympodialis	resistant	+	R1	Ms_res_exp_r1	11116	8434	75.87	
M. sympodialis	resistant	+	R2	Ms_res_exp_r2	27934	23370	83.66	80.11
M. sympodialis	resistant	+	R3	Ms_res_exp_r3	11066	8940	80.79	
M. sympodialis	resistant	-	R1	Ms_res_no_exp_r1	19243	16338	84.9	
M. sympodialis	resistant	-	R2	Ms_res_no_exp_r2	47382	35694	75.33	80.45
M. sympodialis	resistant	-	R3	Ms_res_no_exp_r3	25349	20561	81.11	
M. sympodialis	susceptible	+	R1	Ms_sus_exp_r1	11981	9798	81.78	
M. sympodialis	susceptible	+	R2	Ms_sus_exp_r2	24310	19179	78.89	80
M. sympodialis	susceptible	+	R3	Ms_sus_exp_r3	11261	8934	79.34	
M. sympodialis	susceptible	-	R1	Ms_sus_no_exp_r1	14839	12482	84.12	
M. sympodialis	susceptible	-	R2	Ms_sus_no_exp_r2	13498	11130	82.46	83.71
M. sympodialis	susceptible	-	R3	Ms_sus_no_exp_r3	24309	20553	84.55	
M. sympodialis	resistant	+	R1	Ms_ref_exp_r1	65053	62874	96.65	
M. sympodialis	resistant	+	R2	Ms_ref_exp_r2	41817	39138	93.59	94.18
M. sympodialis	resistant	+	R3	Ms_ref_exp_r3	35182	32472	92.3	
M. sympodialis	resistant	-	R1	Ms_ref_no_exp_r1	24904	23096	92.74	
M. sympodialis	resistant	-	R2	Ms_ref_no_exp_r2	31678	29843	94.21	93.42
M. sympodialis	resistant	-	R3	Ms_ref_no_exp_r3	36836	34367	93.3	
M. furfur	resistant	+	R1	Mf_ref_exp_r1	59633	57781	96.89	
M. furfur	resistant	+	R2	Mf_ref_exp_r2	22327	19276	86.33	92.73
M. furfur	resistant	+	R3	Mf_ref_exp_r3	20146	19134	94.98	
M. furfur	resistant	-	R1	Mf_ref_no_exp_r1	21030	20125	95.70	
M. furfur	resistant	-	R2	Mf_ref_no_exp_r2	13819	12820	92.77	95.34
M. furfur	resistant	-	R3	Mf_ref_no_exp_r3	63533	61982	97.56	
M. furfur	resistant	+	R1	Mf_res_exp_r1	22010	20564	93.43	
M. furfur	resistant	+	R2	Mf_res_exp_r2	67211	63786	94.90	94.07
M. furfur	resistant	+	R3	Mf_res_exp_r3	14739	13835	93.87	

M. furfur	resistant	-	R1	Mf_res_no_exp_r1	45972	43905	95.50	
M. furfur	resistant	-	R2	Mf_res_no_exp_r2	16291	15115	92.78	93.63
M. furfur	resistant	-	R3	Mf_res_no_exp_r3	36156	33488	92.62	
M. furfur	susceptible	+	R1	Mf_sus_exp_r1	54425	53316	97.96	
M. furfur	susceptible	+	R2	Mf_sus_exp_r2	31363	30308	96.64	97.29
M. furfur	susceptible	+	R3	Mf_sus_exp_r3	35332	34367	97.27	
M. furfur	susceptible	-	R1	Mf_sus_no_exp_r1	57264	56061	97.90	
M. furfur	susceptible	-	R2	Mf_sus_no_exp_r2	49726	48571	97.68	98.09
M. furfur	susceptible	-	R3	Mf_sus_no_exp_r3	87067	85931	98.70	

[1]Amphotericin B

Table S3. Exploratory functional enrichment analysis of DEGs in *M. sympodialis* and *M. furfur*.

Species	Gene set	Pathway/biological process	Gene-set size	Expected count	Enrichment ratio	*P* value	Adjusted *P* value (FDR)
M. sympodialis	sce04111	Cell cycle	130	0.36887	5.4220	0.048612	1.00000
M. sympodialis	sce03410	Base excision repair	25	0.07094	14.0970	0.068897	1.00000
M. sympodialis	sce00020	Citrate cycle	31	0.08796	11.3690	0.084813	1.00000
M. sympodialis	sce03020	Transcription	31	0.08796	11.3690	0.084813	1.00000
M. sympodialis	sce01210	2-Oxocarboxylic acid metabolism	42	0.11917	8.3912	0.113390	1.00000
M. sympodialis	sce03420	Nucleotide excision repair	49	0.13904	7.1924	0.131170	1.00000
M. sympodialis	sce04144	Endocytosis	79	0.22416	4.4611	0.203960	1.00000
M. sympodialis	sce01200	Carbon metabolism	112	0.31779	3.1467	0.277940	1.00000
M. sympodialis	sce04011	MAPK signaling pathway	114	0.32347	3.0915	0.282220	1.00000
M. sympodialis	sce01230	Biosynthesis of amino acids	125	0.35468	2.8194	0.305410	1.00000
M. furfur	sce00630	Glyoxylate and dicarboxylate metabolism	29	0.12931	15.4670	0.006874	0.76984
M. furfur	sce00785	Lipoic acid metabolism	12	0.05351	18.6890	0.052327	1.00000
M. furfur	sce00640	Propanoate metabolism	13	0.05797	17.2520	0.056574	1.00000
M. furfur	sce04138	Autophagy	90	0.40130	4.9838	0.058445	1.00000
M. furfur	sce00730	Thiamine metabolism	18	0.08026	12.4600	0.077546	1.00000
M. furfur	sce01200	Carbon metabolism	112	0.49939	4.0049	0.085984	1.00000
M. furfur	sce03060	Protein export	22	0.09810	10.1940	0.094018	1.00000
M. furfur	sce04136	Autophagy	23	0.10255	9.7510	0.098094	1.00000
M. furfur	sce00680	Methane metabolism	26	0.11593	8.6259	0.110220	1.00000
M. furfur	sce00260	Amino Acid metabolism	30	0.13377	7.4758	0.126160	1.00000

Table S4. Statistical analysis of lipid species in amphotericin B-susceptible and resistant *Malassezia* isolates.

	Compound name	Fold change	VIP	*p*-value	*p*-FDR
M. sympodialis[1]	ST 28:1;O6	1.645	0.800	3.23E-02	4.05E-02
	Cyclo-lanostane skeleton	0.655	1.209	4.89E-05	2.82E-04
	Petroselaidic acid	2.585	1.056	1.06E-03	3.22E-03
	NA 27:4;O	0.717	1.061	1.33E-03	3.68E-03
	LDGTS 16:0	0.000	0.929	6.94E-03	1.19E-02
	Sorbitan oleate	0.255	0.851	1.30E-02	1.97E-02
	C17 Sphinganine	0.817	0.869	1.99E-02	2.83E-02
	Margaroyl-EA	0.821	0.832	2.77E-02	3.65E-02
	6-Hydroxy-4,7,22-ergostatrien-3-one	3.367	1.351	1.43E-08	3.65E-07
	Anandamide (20:l, n-9)	1.825	0.751	3.71E-02	4.43E-02
	Basilimoside	0.816	0.814	2.55E-02	3.43E-02
	25-azavitamin D3 / 25-azacholecalciferol	0.787	0.942	8.22E-03	1.38E-02
	taxa-4,11-diene	0.635	0.795	3.53E-02	4.26E-02
	FOH 20:2	0.560	1.129	4.69E-04	1.68E-03
	1,2-Dipalmitoyl-3-succinylglycerol	20.148	1.353	3.76E-08	6.74E-07
	Behenoyl-EA	0.687	0.791	3.13E-02	4.00E-02
	11,13-hexadecadienal	1.773	0.983	4.22E-03	7.83E-03
	Stearolic acid	0.596	0.749	4.06E-02	4.78E-02
	3-pentadec-8-en-1-yl phenol	0.290	0.814	2.59E-02	3.46E-02
	PG O-38:5	3.216	1.183	1.18E-04	5.54E-04
	DG 31:2	1.426	1.166	1.32E-04	5.78E-04
	PC 31:1	0.057	1.159	1.26E-04	5.78E-04
	MG a-25:0	0.000	1.162	2.62E-04	1.07E-03
	7-dehydro-desmosterol	0.766	0.903	1.14E-02	1.79E-02
	DG 32:1	1.569	1.137	4.55E-04	1.66E-03
	HexCer 34:1;O2	3.700	1.263	8.12E-06	6.92E-05
	Zymosterone	0.672	0.948	6.85E-03	1.19E-02
	PG O-38:6	3.057	1.047	1.90E-03	4.52E-03
	DG 33:3	0.503	0.745	4.80E-02	5.44E-02
	Pentacosapentaene	0.552	1.074	1.34E-03	3.68E-03
	Cer 34:1;O2	0.627	1.033	2.48E-03	5.55E-03
	SM 32:1;O2	0.000	0.977	4.19E-03	7.83E-03
	Cer 34:3;O2	0.587	0.800	3.00E-02	3.89E-02
	DG 31:0	0.516	1.024	2.84E-03	6.12E-03
	PC O-36:1	3.387	1.318	3.98E-07	5.47E-06
	PC 35:3	0.000	0.884	1.49E-02	2.22E-02
	FAHFA 34:1;O	0.000	1.078	9.68E-04	3.08E-03
	Palmitoleic acid	0.074	1.077	9.55E-04	3.08E-03
	3-(15-methyl-hexadecanoyloxy)-13-methyl-tetradecanoyl-serine	0.391	0.930	8.47E-03	1.38E-02

Gondoyl-linoleate	0.390	0.930	8.40E-03	1.38E-02
DG 39:5	0.000	1.154	2.40E-04	1.00E-03
FA 18:4;O	0.332	0.953	6.82E-03	1.19E-02
Linoleic acid	0.000	1.061	1.30E-03	3.68E-03
2,4-octadecadienoic acid	0.000	1.134	3.22E-04	1.28E-03
DG 33:2	0.367	1.037	2.33E-03	5.32E-03
Pinolenic acid	0.325	0.915	1.02E-02	1.63E-02
MG 18:1	2.837	1.228	3.65E-05	2.18E-04
DG 33:0	0.323	1.012	3.03E-03	6.25E-03
dolichyl diphosphate	0.085	0.789	3.25E-02	4.05E-02
FAHFA 32:1;O	0.552	0.736	4.75E-02	5.42E-02
FAHFA 32:0;O	0.034	1.352	5.38E-08	8.76E-07
Palmitic acid	0.104	1.317	7.69E-07	8.60E-06
FAHFA 32:0;O	0.031	1.248	1.87E-05	1.39E-04
Elaidolinolenic acid	0.308	1.137	3.47E-04	1.35E-03
Undecylenic acid	0.000	1.311	9.92E-07	1.04E-05
Cer 33:0;O2	0.213	0.793	3.18E-02	4.04E-02
Hydrocortisone butyrate propionate	0.030	1.233	3.09E-05	1.98E-04
Oleic acid	0.120	1.219	3.09E-05	1.98E-04
Myristoleyl arachidonate	0.000	1.128	4.28E-04	1.60E-03
Arachidonoyldihydrosphingosine	0.000	0.764	3.75E-02	4.44E-02
HexCer 39:2;O2	5.807	1.347	2.20E-08	4.93E-07
Cer 36:1;O3	0.444	0.875	1.35E-02	2.04E-02
HexCer 39:1;O3	0.191	0.850	1.77E-02	2.53E-02
Cholest-5-ene	0.399	1.090	1.15E-03	3.42E-03
Cer 35:0;O2	0.234	1.176	2.00E-04	8.52E-04
PE 36:1	1.720	0.809	3.54E-02	4.26E-02
Tetradecahydrocyclopenta[a]phenanthrene	0.000	1.091	9.37E-04	3.08E-03
1-O-Palmitoyl-2-O-linoleoyl-glycerol	0.354	0.718	4.60E-02	5.32E-02
Oleoyl ethanolamine	0.000	0.886	1.24E-02	1.92E-02
4-Hydroxy-16,18-tritriacontanedione	0.000	1.241	1.50E-05	1.17E-04
Cetyl recinoleate	0.442	1.036	2.35E-03	5.32E-03
FA 17:0;O	0.068	0.832	2.65E-02	3.51E-02
DG 33:1	0.285	1.056	1.74E-03	4.26E-03
Cholesteryl caprylate	0.261	1.063	1.58E-03	4.05E-03
FA 18:2	0.275	1.292	3.77E-06	3.55E-05
DG dO-30:0	0.000	1.347	3.75E-08	6.74E-07
Cer 36:1;O2	0.310	0.901	1.27E-02	1.94E-02
Cer 38:0;O2	0.382	1.030	1.89E-03	4.52E-03
DG 29:2	0.411	1.277	7.06E-06	6.32E-05
HexCer 42:0;O2	0.000	1.334	4.29E-07	5.48E-06
MG O-18:1;O	2.828	0.980	5.81E-03	1.05E-02
DG 36:1	3.462	1.079	1.37E-03	3.71E-03
MG 18:1	2.941	1.007	4.03E-03	7.67E-03
22:0-Glc-Campesterol	0.000	1.359	1.15E-08	3.43E-07

	FA 18:4;O	0.000	1.272	9.83E-06	8.00E-05
	SM 44:0;O2	4.369	1.194	6.07E-05	3.37E-04
	FAHFA 32:1;O	0.142	1.407	2.12E-13	3.80E-11
	Cer 46:0;O3	0.006	1.376	5.76E-10	4.12E-08
	Cholesterol	0.000	0.989	3.87E-03	7.53E-03
	FAHFA 32:0;O iso 2	0.095	1.381	1.62E-09	7.25E-08
	FAHFA 34:1;O iso 2	0.597	1.197	9.40E-05	4.68E-04
M. furfur[2]	ST 28:1;O6	0.4	1.05	3.92E-03	9.63E-03
	LPC 18:2	0.7	1.10	2.57E-03	6.94E-03
	Cyclo-lanostane skeleton	1.5	1.03	4.75E-03	1.06E-02
	LPC 16:1	0.6	1.11	2.35E-03	6.70E-03
	NA 27:4;O	1.5	1.01	5.75E-03	1.17E-02
	LDGTS 16:0	0.3	0.89	1.62E-02	2.63E-02
	6-Hydroxy-4,7,22-ergostatrien-3-one	0.4	1.14	2.00E-03	6.11E-03
	Anandamide (20:l, n-9)	2.1	1.36	5.22E-05	3.84E-04
	25-hydroxycholecalciferol	1.2	0.94	1.25E-02	2.11E-02
	1,2-Dipalmitoyl-3-succinylglycerol	3.2	0.97	8.76E-03	1.63E-02
	11,13-hexadecadienal	0.7	0.89	1.62E-02	2.63E-02
	DG 27:0	1.9	1.17	4.93E-04	2.28E-03
	DG 29:1	1.9	1.03	3.83E-03	9.63E-03
	3-pentadec-8-en-1-yl phenol	0.6	0.96	2.57E-02	3.96E-02
	Squamocin K	1.3	0.96	1.19E-02	2.07E-02
	DG 31:2	1.6	1.03	3.87E-03	9.63E-03
	PC 31:1	0.1	0.98	8.92E-03	1.64E-02
	MG a-25:0	5.0	1.37	2.04E-06	2.20E-05
	DG 29:0	1.8	1.09	2.05E-03	6.15E-03
	7-dehydro-desmosterol	1.3	1.17	2.36E-03	6.70E-03
	DG 31:1	1.6	1.01	5.09E-03	1.08E-02
	DG 33:4	1.4	1.14	2.47E-03	6.78E-03
	FA 18:2;O	1.4	1.15	1.10E-03	3.76E-03
	DG 39:4	3.0	1.20	8.85E-04	3.41E-03
	DG 32:1	1.6	1.09	2.45E-03	6.78E-03
	DG 33:3	1.9	1.26	6.62E-04	2.82E-03
	DG 31:0	1.5	1.25	3.62E-04	1.78E-03
	9,10-EpODE	1.4	1.10	2.66E-03	7.06E-03
	Mogrol	1.4	1.19	9.30E-04	3.50E-03
	Cer 34:1;O2	3.2	1.47	2.85E-08	9.91E-07
	SM 32:1;O2	1.8	0.84	3.26E-02	4.94E-02
	Cer 34:3;O2	2.9	1.47	1.25E-07	2.88E-06
	DG 31:0	2.8	1.42	1.02E-06	1.37E-05
	PC 35:3	0.5	0.90	3.43E-02	5.15E-02
	FAHFA 34:1;O	2.9	1.40	4.86E-06	4.64E-05
	Palmitoleic acid	2.9	1.42	1.32E-06	1.64E-05
	3-(15-methyl-hexadecanoyloxy)-13-methyl-tetradecanoyl-L-serine	1.9	1.31	1.53E-04	9.17E-04

Gondoyl-linoleate	1.9	1.32	1.46E-04	9.08E-04
FA 18:4;O	2.4	1.41	4.35E-06	4.40E-05
Linoleic acid	1.8	1.18	1.67E-03	5.19E-03
2,4-octadecadienoic acid	1.7	1.11	3.02E-03	7.89E-03
DG 33:2	1.8	1.25	3.45E-04	1.75E-03
Pinolenic acid	1.8	1.17	2.28E-03	6.70E-03
DG 33:0	2.0	1.23	6.23E-04	2.73E-03
PC 32:0	2.7	1.15	7.19E-04	2.98E-03
FAHFA 32:1;O	0.5	1.13	6.19E-04	2.73E-03
FAHFA 32:0;O	2.0	1.06	4.41E-03	1.03E-02
Palmitic acid	1.9	1.27	2.19E-04	1.27E-03
FAHFA 32:0;O	2.1	1.18	1.05E-03	3.76E-03
Elaidolinolenic acid	1.5	1.20	1.11E-03	3.76E-03
Undecylenic acid	1.6	0.98	1.16E-02	2.04E-02
Cer 33:0;O2	2.5	1.11	3.86E-03	9.63E-03
FAHFA 34:1;O	1.5	1.01	9.47E-03	1.72E-02
Hydrocortisone butyrate propionate	2.2	1.19	8.83E-04	3.41E-03
Oleic acid	1.4	1.07	4.86E-03	1.06E-02
Myristoleyl arachidonate	1.6	1.29	2.31E-04	1.29E-03
Arachidonoyldihydrosphingosine	2.7	1.34	7.32E-05	5.16E-04
Cer 35:0;O2	2.6	1.50	8.62E-10	1.40E-07
PE 36:1	1.7	0.86	1.82E-02	2.92E-02
Oleoyl ethanolamine	1.5	0.94	2.50E-02	3.89E-02
4-Hydroxy-16,18-tritriacontanedione	2.4	1.25	1.21E-04	8.16E-04
Cer 36:0;O2	2.0	1.20	3.35E-04	1.75E-03
FA 17:0;O	4.7	1.45	9.65E-07	1.37E-05
DG 33:1	1.2	0.97	2.19E-02	3.45E-02
FA 18:2	1.3	1.03	7.62E-03	1.47E-02
DG dO-30:0	1.3	1.02	7.09E-03	1.40E-02
Cer 36:1;O2	3.2	1.39	6.18E-06	5.56E-05
Cer 38:0;O2	2.5	1.44	1.00E-06	1.37E-05
DG 29:0	1.6	1.01	5.36E-03	1.12E-02
TG 53:5	3.7	1.25	3.11E-04	1.68E-03
DG 29:2	1.8	1.15	7.89E-04	3.20E-03
DG 31:1	1.4	0.99	6.75E-03	1.35E-02
22:0-Glc-Campesterol	2.0	1.07	4.23E-03	1.01E-02
FA 18:4;O	3.0	1.13	1.49E-03	4.92E-03
SM 44:0;O2	0.0	1.02	1.35E-02	2.23E-02
Cer 46:0;O3	2.8	1.19	4.33E-04	2.06E-03
FAHFA 32:0;O iso 2	2.1	0.95	1.16E-02	2.04E-02
FAHFA 34:1;O iso 2	1.3	1.04	4.53E-03	1.03E-02

[1]*M. sympodialis* susceptible to amphotericin B vs *M. sympodialis* resistant to amphotericin B + *M. sympodialis* reference

[2]*M. furfur* susceptible to amphotericin B vs *M. furfur* resistant to amphotericin B + *M. furfur* reference

Table S5. Relative abundance of DLMs by class when comparing *Malassezia* spp. susceptible and resistant to amphotericin B.

Lipid name	Relative abundance (Avg.)			
	MfurS[1]	MfurR[2]	MsymS[3]	MsymR[4]
**Fatty acids**				
Palmitic acid	0.496817627	0.309420798	0.047900253	0.465975919
Palmitoleic acid	0.255655518	0.10445202	0.011321522	0.157263186
11,13-hexadecadienal	0.341728169	0.627809938	0.453479474	0.263140536
FA 17:0;O	0.454906141	0.126092275	0.005406423	0.08335998
Oleic acid	0.420004714	0.356962825	0.030128709	0.255037663
Linoleic acid	0.064866584	0.044037421	0	0.036351627
2,4-octadecadienoic acid	0.044683074	0.030114426	0	0.026587775
FA 18:2	0.14961436	0.139737061	0.03932019	0.144549704
FA 18:2;O	0.548919906	0.474956675	0.452883478	0.403031606
FA 18:4;O	0.051875916	0.017942369	0	0.050477763
FA 18:4;O	0.02830074	0.013771586	0.007535937	0.023243668
9,10-EpODE	0.192233721	0.159465395	0.129530085	0.145994366
NA 27:4;O	0.034742661	0.027064012	0.011826618	0.016468454
FAHFA 32:0;O	0.130699532	0.072341923	0.005059846	0.148817495
FAHFA 32:0;O	0.707903627	0.374532371	0.025773487	0.850548001
FAHFA 32:0;O iso 2	0.38260284	0.193642518	0.033069956	0.346608331
FAHFA 32:1;O	0.018993087	0.049537828	0.007534226	0.013984974
FAHFA 32:1;O	0.422245407	0.296542667	0.052871295	0.371152409
FAHFA 34:1;O	0.0450832	0.01830725	0	0.028159494
FAHFA 34:1;O	0.059144056	0.048112229	0.002771155	0.033696945
FAHFA 34:1;O iso 2	0.08174139	0.073262518	0.037794029	0.06303893
Gondoyl-linoleate	0.123479838	0.079111905	0.028304676	0.07453045
**Glycerolipids**				
MG 18:1	0.01018665	0.01107748	0.08863147	0.02895379
MG 18:1	0.0088157	0.01354806	0.06320741	0.02134303
MG O-18:1;O	0.01075342	0.01152525	0.08508242	0.02894974
MG a-25:0	0.03536466	0.00904878	0	0.03348248
DG 27:0	0.02535846	0.01548642	0.01953743	0.01608099
DG 29:0	0.08913745	0.06285455	0.03348204	0.08962414
DG 29:0	0.10743232	0.06849383	0.13331172	0.11355082
DG 29:1	0.10593434	0.063336	0.07077796	0.0779347
DG 29:2	0.0446107	0.02877021	0.01705975	0.04112162
DG 31:0	0.27299239	0.11099486	0.12730733	0.25113904
DG 31:0	0.40950161	0.31820021	0.25934004	0.33226502
DG 31:1	0.0891887	0.07303038	0.04205513	0.07168935
DG 31:1	0.80089138	0.5710273	0.72924788	0.61118929
DG 31:2	0.15579414	0.11326991	0.14217889	0.09925993
DG 32:1	0.1072924	0.0940833	0.10688018	0.06708073
DG 33:0	0.03465194	0.02076462	0.00966565	0.03079904
DG 33:1	0.37498267	0.36633616	0.08442073	0.30305262
DG 33:2	0.08802293	0.05766045	0.03130939	0.08641946
DG 33:3	0.04991295	0.03264651	0.02601618	0.05337711

Lipid name	Relative abundance (Avg.)			
	MfurS[1]	MfurR[2]	MsymS[3]	MsymR[4]
DG 33:4	0.03743656	0.03090665	0.04100395	0.04648971
1-O-Palmitoyl-2-O-linoleoyl-glycerol	0.10031812	0.14761411	0.06242506	0.16922482
DG 36:1	0.07205013	0.07566129	0.75704832	0.21007885
DG 39:4	0.03425368	0.01610591	0	0.05124721
DG 39:5	0.01623491	0.01552496	0	0.0089181
DG dO-30:0	0.05050396	0.04483355	0	0.04540694
TG 53:5	0.00196017	0.00053676	0	0.00061675
LDGTS 16:0	0.09420912	0.40892522	0	0.2909907
**Glycerophospholipids**				
PC 31:1	0.00455556	0.04308566	0.00061119	0.01082908
PC 32:0	0.02823442	0.01353599	0.00105502	0.00171214
PC 35:3	0.00189448	0.00449992	0	0.00326135
PE 36:1	0.01760023	0.01164059	0.04930689	0.02785504
LPC 16:1	0.1037872	0.23678289	0.14424571	0.20738451
LPC 18:2	0.26340688	0.44606747	0.55540726	0.50017365
PC O-36:1	0.00071723	0.00128831	0.01224448	0.00349473
PG O-38:5	0.00111044	0.002055	0.00419501	0.00122389
PG O-38:6	0.00064108	0.00127945	0.00250649	0.00076879
**Sphingolipids**				
Cer 33:0;O2	0.013466126	0.00685443	0.0017854	0.00874577
Cer 36:1;O2	0.091783862	0.03714966	0.00915611	0.03035974
Cer 34:3;O2	0.2428067	0.10145656	0.0783948	0.13703101
Cer 35:0;O2	0.047101595	0.02174865	0.00587476	0.02537116
Cer 36:0;O2	0.08466797	0.04750387	0.0385621	0.04147196
Cer 36:1;O2	0.091783862	0.03714966	0.00915611	0.03035974
Cer 36:1;O3	0.05862953	0.12231223	0.0252213	0.05538169
Cer 38:0;O2	0.123181109	0.06231126	0.01351243	0.03592793
Cer 46:0;O3	0.417022063	0.16162778	0.00245304	0.40757094
HexCer 34:1;O2	0.022545003	0.0469234	0.06714338	0.01701601
HexCer 39:1;O3	0.005715552	0.01397945	0.0010816	0.00586889
HexCer 39:2;O2	0.000804975	0.00110151	0.00423727	0.00068273
HexCer 42:0;O2	0.004365169	0.00904668	0	0.01136363
SM 32:1;O2	0.019019266	0.01141574	0	0.03804783
SM 44:0;O2	0	0.00172544	0.00778628	0.00166951
3-(15-methyl-hexadecanoyloxy)-13-methyl-tetradecanoyl-Serine	0.123716616	0.07930161	0.02838266	0.07467579
**Sterols**				
Zymosterone	0.04108861	0.04321984	0.02050175	0.03083671
22:0-Glc-Campesterol	0.00469393	0.00266642	0	0.00322927
25-hydroxycholecalciferol	0.01753913	0.01747289	0.01171964	0.01342055
25-azavitamin D3/25-azacholecalciferol	0.1142774	0.12389921	0.06929183	0.08873871
6-Hydroxy-4,7,22-	0.01933162	0.06210832	0.10062424	0.02915047

Lipid name	Relative abundance (Avg.)			
	MfurS[1]	MfurR[2]	MsymS[3]	MsymR[4]
ergostatrien-3-one				
7-dehydro-desmosterol	0.09977316	0.09576845	0.05434084	0.0715726
Cholest-5-ene	0.01699456	0.02115574	0.00600546	0.01530249
Cholesterol	0.10085198	0.1078777	0	0.10146756
Cyclo-lanostane skeleton	0.04330392	0.03318221	0.01312618	0.02000671
ST 28:1;O6	0.05792824	0.1842149	0.3668245	0.2273591
Tetradecahydrocyclopenta[a] Phenanthrene	0.03093551	0.05529659	0	0.05390052
Dolichyl diphosphate	0.00584326	0.00473899	0.00174272	0.02165146

[1]*M. furfur* susceptible to amphotericin B, [2]*M. furfur* resistant to amphotericin B + *M. furfur* CBS 1878, [3]*M. sympodialis* susceptible to amphotericin B, [4]*M. sympodialis* resistant to amphotericin B + *M. sympodialis* CBS 7222.
